# Genetic analysis of a phenotypic loss in the mechanosensory entrainment of a circalunar clock

**DOI:** 10.1371/journal.pgen.1010763

**Published:** 2023-06-22

**Authors:** Dušica Briševac, Celine Prakash, Tobias S. Kaiser

**Affiliations:** Max Planck Institute for Evolutionary Biology, Max Planck Research Group Biological Clocks, Plön, Germany; Universidad de Valparaiso, CHILE

## Abstract

Genetic variants underlying traits that become either non-adaptive or selectively neutral are expected to have altered evolutionary trajectories. Uncovering genetic signatures associated with phenotypic loss presents the opportunity to discover the molecular basis for the phenotype in populations where it persists. Here we study circalunar clocks in populations of the marine midge *Clunio marinus*. The circalunar clock synchronizes development to the lunar phase, and it is set by moonlight and tidal cycles of mechanical agitation. Two out of ten studied populations have lost their sensitivity to mechanical agitation while preserving sensitivity to moonlight. Intriguingly, the F1 offspring of the two insensitive populations regained the sensitivity to mechanical entrainment, implying a genetically independent loss of the phenotype. By combining quantitative trait locus mapping and genome-wide screens, we explored the genetics of this phenotypic loss. QTL analysis suggested an oligogenic origin with one prevalent additive locus in one of the strains. In addition, it confirmed a distinct genetic architecture in the two insensitive populations. Genomic screens further uncovered several candidate genes underlying QTL regions. The strongest signal under the most prominent QTL contains a duplicated *STAT1* gene, which has a well-established role in development, and *CG022363*, an ortholog of the *Drosophila melanogaster CG32100* gene, which plays a role in gravitaxis. Our results support the notion that adaptive phenotypes have a complex genetic basis with mutations occurring at several loci. By dissecting the most prevalent signals, we started to reveal the molecular machinery responsible for the entrainment of the circalunar clock.

## Author summary

Biological clocks allow organisms to synchronize their physiology and behaviour with their environment. A key part of this mechanism are reliable environmental cues that play a role in setting the clock. Here we study the circalunar clock of *Clunio marinus*, a marine insect that inhabits the intertidal zone. The circalunar clock is set by moonlight as well as mechanical agitation caused by the tides. Interestingly, in two out of ten studied *Clunio* populations, mechanical agitation was not sufficient to set the circalunar clock. We found that this loss of sensitivity has a genetic basis. Furthermore, we use the insensitive populations as natural mutants. Finding the mutations that lead to the loss of sensitivity allows us to uncover the molecular mechanisms underlying the setting of the yet unexplored molecular clockwork. Additionally, by uncovering the genetic basis for the loss of sensitivity in two independent populations, we get insights into the processes underlying the evolutionary adaptation of biological clocks.

## Introduction

Life on earth adapted to anticipate predictable changes in its environment in order to survive, a case in point is the ubiquity of biological clocks. Due to the earth’s rotation around its axis, most living creatures are exposed to 24-hour cycles, which has resulted in the pervasiveness of circadian clocks [1,2]. Furthermore, marine organisms inhabiting intertidal zones are exposed to tidal cycles of 12.4 hours (or sometimes 24.8 hours), which are modulated across the 29.53-day lunar cycle. Thus, marine organisms have evolved circatidal and circalunar clocks. Due to their universal occurrence, circadian clocks have been intensely studied over the last century [3,4]. Comparatively much less is known about circatidal and circalunar clocks [5–10], although some argue that as life evolved in the marine environment circadian clocks may have evolved from evolutionarily older circatidal or circalunar clocks [11,12].

Biological clocks must be appropriately set to fulfill their role in synchronizing endogenous physiological processes, reproduction, and behavior to the exogenous environmental cycles. Environmental variables that reliably fluctuate with geophysical cycles serve as clock synchronizers, so-called zeitgebers. The most studied zeitgeber is the light-dark cycle that synchronizes the circadian clock [1]. Two other synchronizers of the circadian clock that were experimentally confirmed are temperature and vibration [13–16]. In contrast, many environmental variables fluctuate with the tides and the following have been shown to serve as strong zeitgebers of the tidal clocks: mechanical disturbance of the water [17–19], changes in hydrostatic pressure [18,20,21], temperature fluctuations [22,23], changes in salinity [24] immersion and emersion [22].

Not surprisingly, moonlight was shown to be a unique cue for synchronizing lunar clocks [25–29]. Furthermore, several synchronizers that were first discovered as tidal cues, were consequently demonstrated to be strong zeitgebers for setting circalunar clocks: vibration that accompanies the rise and fall of the tides [30,31] and temperature fluctuations [32]. Depending on the stability and robustness of the cycles in the environment that the organism inhabits, different zeitgebers provide reliable cues for biological clocks in different organisms. Finally, while biological clocks are not crucial for the survival of all organisms, the harsher the environmental cycles, the stronger the selection pressure on the presence of reliable biological clocks. Studying organisms inhabiting these harsh environments promises to give insight into the nature of yet unexplored biological clocks. One such species whose survival critically depends on its ability to simultaneously synchronize to lunar and circadian cycles is the marine midge *Clunio marinus*.

*Clunio* spends most of its life in a larval stage submerged in the intertidal zone of the Atlantic Ocean. During full moon and new moon, adults emerge on the sea surface, mate, oviposit eggs and die within a few hours. Circadian and circalunar clocks allow them to precisely time reproduction to the lowest of the low tides. Individuals that do not emerge at the appropriate time miss the ecologically suitable low tide for reproduction and the opportunity to mate and are thus eliminated from the population. Therefore, strong selection pressure shapes various timing phenotypes in populations that encounter different tidal regimes along the Atlantic coast [33–37]. Moonlight, tidal turbulence and temperature have been shown to be zeitgebers setting the circalunar clock of *Clunio marinus* [27,31,32,38]. However, different *Clunio* populations are differentially sensitive to zeitgebers, most likely due to the unreliability of different zeitgebers in certain geographical locations [38]. Neumann discovered one population insensitive to moonlight and two that were insensitive to tidal turbulence [38]. Tidal turbulence was defined as low frequency, low amplitude vibration that coincides with the rising tide [38]. This stimulus shifts every day by 50 minutes resulting in a semi-lunar 14.7 days entrainment pattern [38].

Evolutionary losses of function can have a creative role in evolution [39], and genetic and genomic analysis of the affected populations can identify the genes involved in corresponding molecular pathways [40]. Our goal was to establish if the loss of mechanosensory entrainment in the two populations was consistent with it having a common genetic basis, or whether it occurred independently in each population. We also sought to determine if genetic control of this phenotype is likely controlled by a single locus of major effect or whether multiple loci play discernible roles. Finally, we aimed to identify genes likely to be responsible for impacting the trait.

## Results

### Loss of sensitivity to mechanical entrainment is a genetically determined trait that evolved independently in two *Clunio* populations

**Fig 1:**
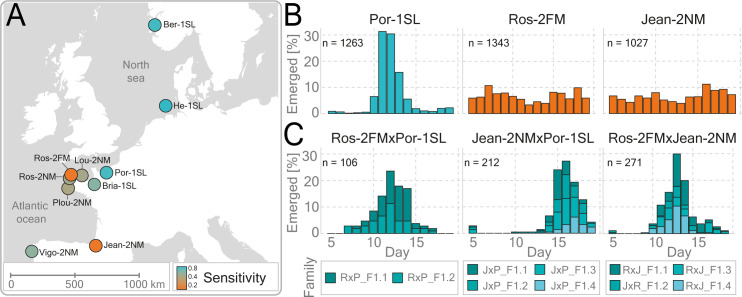
Sensitivity to mechanical entrainment was lost twice independently in European *Clunio* populations. (A) The origin of the ten *Clunio* populations used in this study is shown on the map (S1 Fig). Map data was obtained from https://www.naturalearthdata.com/downloads/50m-physical-vectors/. Heatmap depicts the sensitivity of each strain to mechanical entrainment as estimated by the circular statistics (Table 1). (B, C) Graphs show the fraction of emerged individuals per lunar day upon mechanosensory entrainment. The total number of emerged individuals is depicted in the left corner of each bar graph, and raw data is given in S1 and S2 Tables. (B) Graphs depict the emergence patterns of the parental populations. Ros-2FM and Jean-2NM populations are insensitive to tidal turbulence as shown by the arhythmic emergence patterns, while the Por-1SL population is sensitive. Geographical locations and the years when strains were established are given in S1 Table. (C) Crossing sensitive and insensitive strains resulted in sensitive F1 progeny. Furthermore, when the two insensitive strains were crossed, the resulting F1 hybrids regained sensitivity to the entrainment (right). Individual F1 families that were raised together were depicted in different shades of blue. The total number of individuals per family is listed in S2 Table.

The circalunar clock robustly regulates the emergence of *Clunio* adults over a lunar month. We study this phenomenon under laboratory conditions by counting the number of emerged adults per day over several lunar cycles, and then assess characteristics of the phenotype using circular statistics: phase, period, rhythmicity, etc. The sensitivity of different strains to the zeitgebers is therefore estimated indirectly via the strength of their emergence rhythms upon entrainment to moonlight or tidal turbulence. Strains are named based on an abbreviation for the sampling sites and the time point of emergence under moonlight entrainment: NM = „new moon“, FM = „full moon“, SL = „semi-lunar”= full moon and new moon. Here we tested the entrainment of Plou-2NM, Ros-2NM, Lou-2NM, Bria-1SL, and Por-1SL under tidal turbulence for the first time, while the entrainment to moonlight [35,41,42] and tidal turbulence [38] were previously reported for the other populations (Figs 1A and S1 and Tables 1, S1, and S2). Vigo-2NM is the most southern strain and it is sensitive to tidal turbulence. Going north, we come across Jean-2NM which is insensitive to tidal turbulence, followed by five closely related populations at the coast of Bretagne: Plou-2NM, Ros-2FM, Ros-2NM, Lou-2NM, Bria-1SL, Por-1SL; and finally, the two most northern populations: He-1SL in Germany and Ber-1SL in Norway (Fig 1A). Bretagne populations vary from very sensitive in the north (Por-1SL and Bria-1SL), and intermediate sensitivity in the south (Ros-2NM, Lou-2NM, and Plou-2NM) to completely insensitive (Ros-2FM) (Figs 1A and S1 and Table 1). This suggests that the frequency of the “insensitive alleles” may vary among the Bretagne populations, giving rise to varying degrees of sensitivity. Furthermore, as Ros-2FM and Jean-2NM are arrhythmic under tidal turbulence but rhythmic under moonlight (Figs 1B, S1O, and S1S) [38], we can conclude that their lunar clocks are intact, but sensory inputs have evolved rendering them insensitive to one of the cues. To characterize the genetic basis for this phenotypic loss, we crossed turbulence-insensitive strains to a strain sensitive to both tidal turbulence and moonlight, Por-1SL (Figs 1B, S1F, and S1G), and analyzed the emergence of adults in F1 and F2 generations (Figs 1B, 1C, and S2). We calculated circular statistics for the emergence distributions in Figs 1 and S1, and used vector length of the summary circular statistics for estimating the strength of the entrainment (Table 1). We found that sensitivity to tidal turbulence is genetically determined and a dominant trait (Table 1).

**Table 1:** Ros-2FM and Jean-2NM are insensitive to tidal turbulence.

Circular summary statistic	Ber-1SL	He-2SL	Por-1SL (Sensitive parent)	Bria-2SL	Plou-2NM	Lou-2NM	Ros-2FM (Insensitive parent)	Ros-2NM	Vigo-2NM	Jean-2NM (Insensitive parent)	RxP-F1.1	RxP-F2.1	RxP-F1.2	RPxR-BC.1	JxP-F1.1	JxP-F2.1.6	JxP-F1.2	JxP-F2.2.3
Median vector direction [lunar day]	8	11	12	6	14	2	6	15	13	2	10	10	12	11	15	14	13	13
Mean vector direction [lunar day]	8.25	10.63	11.89	5.99	14.02	1.90	5.44	14.81	12.74	2.38	9.68	9.93	11.52	11.18	0.04	14.04	13.39	12.64
**Vector length**	**0.78**	**0.80**	**0.77**	**0.63**	**0.50**	**0.52**	**0.11**	**0.54**	**0.61**	**0.12**	**0.60**	**0.51**	**0.78**	**0.37**	**0.87**	**0.63**	**0.85**	**0.49**
Circular standard deviation	0.70	0.66	0.73	0.96	1.17	1.14	2.11	1.10	0.99	2.05	1.01	1.16	0.70	1.42	0.52	0.95	0.56	1.19
Angular variance	0.43	0.39	0.47	0.73	0.99	0.96	1.78	0.91	0.78	1.76	0.80	0.98	0.44	1.27	0.25	0.73	0.29	1.02
Angular deviation	0.66	0.62	0.68	0.86	1.00	0.98	1.34	0.96	0.88	1.33	0.89	0.99	0.66	1.13	0.50	0.86	0.54	1.01

We used circular statistics to evaluate sensitivity to tidal turbulence in 10 laboratory strains (Figs 1A and S1 and S1 Table), and four crossing families that were used for QTL mapping. Ros-2FMxPor-1SL cross was abbreviated RxP and Jean-2NMxPor-1SL JxP (the full list is given in S2 Table). For example, “RxP-F1.1” is Ros-2FMxPor-1SL F1 cross family 1, “RPxR-BC1” is (Ros-2FMxPor-1SL)xRos-2FM backcross family 1 and “JxP-F2.1.6” is Jean-2NMxPor-1SL F2 cross family 1.6 (family 6 of a series of crosses that all go back to the same parents). Mean and median vector direction measure lunar phase, while vector length, deviation, and variance correlate with the strength of the rhythm or the sensitivity to the zeitgeber. We used vector length as a phenotypic score for QTL mapping and association mapping (bold).

To test if the same mutations are responsible for the loss of sensitivity in Jean-2NM and Ros-2FM we performed a complementation cross. Interestingly, the four F1 families raised separately all regained their sensitivity to mechanical entrainment (Fig 1C). This finding strongly suggests a different and recessive genetic basis for the loss of sensitivity in Jean-2NM and Ros-2FM.

### Discovering genomic loci responsible for the phenotypic loss in the Ros-2FM population

**Fig 2:**
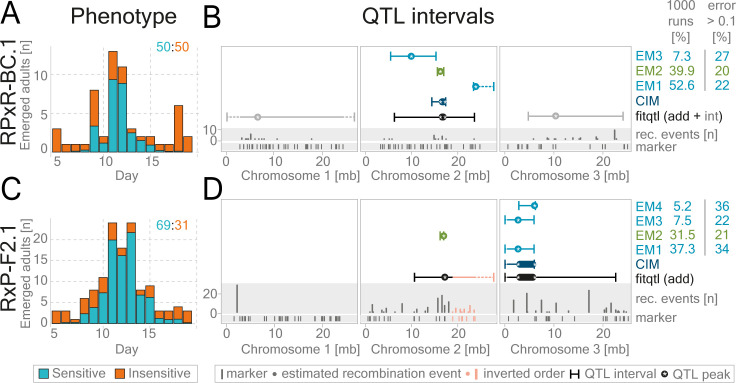
QTL mapping in two Ros-2FmxPor-1SL mapping families reveals one shared additive QTL on the second chromosome. Regions of the genome harboring genes responsible for the loss of mechanical entrainment in the Ros-2FM population were identified in a [Ros-2FM x Por-1SL] x Ros-2FM backcross (RPxR-BC.1), see panels A and B, and a [Por-1SL x Ros-2FM] F2 intercross (RxP-F2.1), see panels C and D. Bar graphs show the number of emerged individuals per day (A, C). The proportion of insensitive (orange) and sensitive (blue) individuals found on each day was calculated based on estimated probabilities (S3 and S4 Figs). The ratio of sensitive and insensitive individuals in each family is indicated in the top right corner. (B, D) QTL intervals are given for: composite interval mapping–dark blue, fitqtl: additive loci–black, fitqtl: epistatic loci–gray, EM-algorithm–light blue. The green marks the phenotypic panel with the highest convergence in EM analysis (i.e. the number of times the panel was found to be the best in 1000 runs) and the lowest error (i.e. the fraction of individuals in each panel for which the binary phenotype differs significantly from the starting probabilities; see Methods QTL mapping/EM-pipeline, S3 Table). Raw data is given in S3 Table.

Quantitative trait loci (QTL) mapping was conducted to locate the regions of the genome containing genetic variants responsible for the loss of sensitivity to tidal turbulence in the Ros-2FM population. The resolution of QTL mapping depends on the number and distribution of markers as well as the recombination events which in turn depends on the number of individuals in the crossing family. To maximize our chances of achieving narrow confidence intervals, we performed a large number of crosses and then selected two families for the analysis: F2 progeny of Ros-2FMxPor-1SL cross (RxP-F2.1) and a backcross progeny of Ros-2FMxPor-1SL F1 female to Ros-2FM male (RPxR-BC.1) (S2 Table). The number of informative markers was 137 in RxP-F2.1 and 123 in RPxR-BC.1. The total number of recombination events was 269 and 61, while the number of unique genomic positions of the recombination events was 51 and 36 in RxP-F2.1 and RPxR-BC.1 families respectively (Fig 2B and 2D).

If ~130 markers and ~40 unique recombination events would be evenly distributed along the 80Mb genome, we could achieve the mapping resolution of ~2-3Mb. However, several non-recombining regions were found in both families and one in which the marker order was inverted as compared to the reference (Fig 2B and 2D). These regions are thought to be large polymorphic inversions [43] which are limiting mapping resolution on the first chromosome and in the right arm of the second chromosome [44].

In order to phenotype F2 and BC progenies, we must distinguish between “sensitive” individuals that emerged within the Por-1SL-like peak and “insensitive” individuals that can emerge on any lunar day. However, the emergence peak does not only contain sensitive individuals, but also some of the insensitive individuals. To overcome this issue, we tested different phenotyping strategies and mapping algorithms (S3–S10 Figs) (see Methods and S1 Methods for more details). We calculated the probability of finding sensitive and insensitive individuals on each lunar day (S3 and S4 Figs) and used it as a phenotypic score for the QTL analysis. In addition, we generated a reduced dataset by excluding the individuals with uncertain phenotypes and treated those with the probability of being „insensitive”higher than 0.7 as „insensitive”and lower than 0.3 as „sensitive”(S3 Fig). Finally, this approach allowed us to estimate the ratio of the two phenotypes in the F2 and BC generations: 69:31 in the RxP-F2.1 intercross (Fig 2C) and 50:50 in the RPxR-BC.1 backcross (Fig 2A). The difference in ratios is attributed to a higher portion of sensitive individuals (parental and F1 genotypes) in an F1xF1 intercross as compared to an F1xRos-2FM backcross. Similar ratios were found in Jean-2NMxPor-1SL intercross families (see below). Such segregation of parental phenotypes in F2 and BC progenies indicates that this trait is determined by a small number of loci.

Furthermore, in order to screen for additive QTLs, we ran standard interval mapping with *scanone* (S6E–S6H Fig) and composite interval mapping (Figs 2B, 2D, and S6I–S6L). To investigate QTLs in epistasis we ran a two-dimensional scan with *scantwo* function (S7 Fig). QTLs identified with *scanone* and *scantwo* were then fed into the multiple-QTL-mapping pipeline implemented in the R/qtl package with the *fitqtl* function (Figs 2B, 2D, S6Q–S6T, and S7). Since various models can be significant with *fitqtl*, we also tested a Bayesian method implemented in R package *qtlbim* designed to find the best QTL model for *fitqtl* (S6Y-AB Fig).

The multiple QTL mapping pipeline revealed one additive QTL and two QTLs in epistasis in the RPxR-BC.1 family, and two additive QTLs in the RxP-F2.1 family (Figs 2B, 2D, and S6–S8). The additive QTL on the second chromosome was found in both crossing families. The QTL on the third chromosome interacts additively with the QTL on the second chromosome in the RxP-F2.1 reduced dataset (S7E and S7F Fig), while in the RPxR-BC.1 family it is in a negative additive-by-additive epistatic interaction [45] with the QTL on the first chromosome. The QTL on the first chromosome has a positive additive effect in the heterozygous AB background of the QTL on the third chromosome and vice-versa (Figs 2D and S7A–S7D). The QTLs in epistasis were found only in one of the families, potentially because the presence of the epistatic interaction depends on the genetic background. This can occur if the mutations underlying QTLs in epistasis are not fixed in the two populations. In other words, if a mutation underlying QTL1 only has an effect in the presence of another mutation underlying QTL2, and one of the two alleles is absent in the parent of that crossing family, the epistatic interaction would not be identified. Thus, to find the regions of the genome containing the loci most likely pervasive in the natural populations, we further focused only on the additive QTLs.

In order to further estimate the effect of the phenotyping uncertainty on additive QTLs, we generated the *scanone*-optimized expectation-maximization pipeline (see Methods for more details, Figs 2 and S3–S8). In a nutshell, all individuals are assigned binary phenotypes (0 or 1) depending on their starting phenotype probabilities. Then the algorithm changes the phenotypes of individuals in order to find the binary phenotype panel with the highest LOD score (Figs 2C, 2D, and S6M–S6P, for more details see Methods). The resulting binary phenotype panels are assessed for their credibility by how often the algorithm converges to a specific panel (% convergence) and by which fraction of animals differs by more than 0.1 to the starting probability. In order to first test this pipeline, we used sex as a known binary phenotype, transformed it into a probability phenotype with varying degrees of uncertainty, and tested how well the QTL intervals from the resulting phenotypic panels match the true sex locus (S9 and S10 Figs). The phenotypic panels with the highest degree of convergence and lowest error match extremely well with the true sex locus (S9 Fig), showing the validity of our approach.

In the RPxR-BC.1 family, the QTL interval of the EM binary panel with the lowest percentage of individuals with error>0.1 (20%) and the second-highest percentage of convergence (39.9%) perfectly overlaps with the QTL interval provided by CIM, and *fitqtl* mapping on probability phenotypes (Figs 2B, S8A, and S8B). In the RxP-F2.1 family, the best panel according to the same criteria is also on the second chromosome: the percentage of convergence is 31.5, percentage of individuals with error > 0.1 is 21% (Figs 2D, 2K, S8C, and S8D). The high level of convergence in the phenotype panels shows that the QTL landscape does not contain too many potential local optima. This suggests that the effect of the uncertainty on the QTL analysis is limited and does not have a major impact on the QTL location.

Other than the phenotyping uncertainty, a polygenic or oligogenic origin could also lead to a reduction in the QTL LOD scores. In the RPxR-BC.1 family, the full *fitqtl* model explains 28% of the phenotypic variance (S6–S8 Figs and S3 Table). In the reduced (binary) dataset that number increases to 40.79% (S6–S8 Figs and S3 Table). The QTL on the second chromosome alone explains 9.27% and the epistatic interaction between the first and the third chromosome explains 11.09%; while in the reduced dataset that becomes 2.82% and 11.68% respectively. In the RxP-F2.1 family, the *fitqtl* model explains 13.74% in the full and 21.8% in the reduced dataset. The percentage of variance explained by the QTL on the second chromosome is 5.9% and on the third chromosome is 8% (18.29% and 16.56% respectively in the reduced dataset). Previous QTL analysis on mapping the lunar phase, a phenotype of discrete nature, identified two QTLs: one explains 23% of the variation and the other 14% [42]. In both the present and the previous study, we find a small number of significant loci impacting a trait, with a comparable proportion of phenotypic variance explained. This potentially indicates that we did not lose significant mapping power due to the non-discrete nature of the phenotype in the current study. Furthermore, this implies that if the detected loci collectively account for up to 20–40% of the phenotypic variance, unidentified loci or loci of small effect size may still play a substantial role.

**Fig 3:**
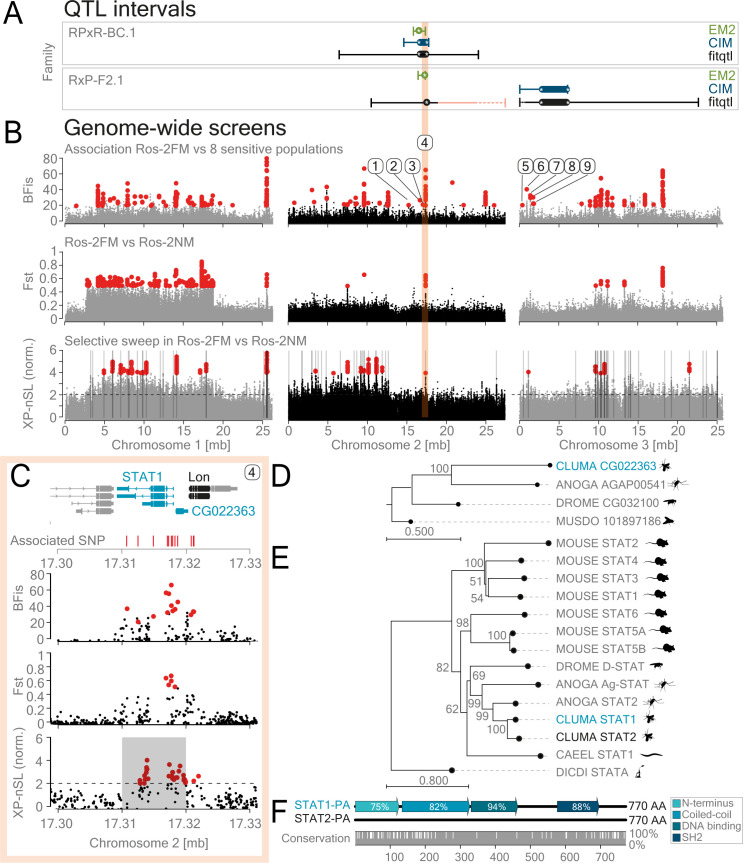
A combination of QTL mapping and genome-wide screens points to *STAT-1* and gravitaxis gene *CG022363* as likely contributing to the loss of sensitivity to mechanical entrainment in the Ros-2FM population. (A) QTL intervals from the two mapping families are plotted along the three chromosomes (modified from Fig 2B and 2D). (B-top). The 746.887 variants called from Ros-2FM and eight differentially sensitive populations were screened for their association with the sensitivity to mechanical entrainment using BayPass. Sensitivity to mechanical entrainment was estimated from emergence patterns using circular statistics (Table 1). Median vector length was used as the phenotypic score. The Bayesian factor (BFis) depicting the strength of the association is plotted for all the variants along the three chromosomes (gray and black). 375 significantly different variants, as determined by BFis > = 20, eBPis > = 2, XtXst > = 21.67 are marked in red (see Methods section for details, raw data are published on Max Planck Repository Edmond https://doi.org/10.17617/3.HUYLPR). The numbers indicate loci within the QTL intervals. Zoom into locus 4 is shown in panel (C) and the others in S12 Fig. (B-middle and bottom plots): To expose potentially adaptive genetic variants under positive selection as a result of local adaptation, we contrasted the turbulence-insensitive Ros-2FM population with the sympatric Ros-2NM population, determined to be sensitive to this entrainment (S1 Fig). (B-middle). Genetic differentiation (F_ST_) was plotted for the variants found in Ros-2FM and Ros-2NM populations. Red marks F_ST_ values above 0.5. (B-bottom). The cross-population nSL (number of segregating sites by length) statistic shows the decay of haplotype homozygosity surrounding adaptive alleles as a result of a selective sweep in Ros-2FM in contrast to Ros-2NM. The top 1% clusters of at least 10 variants with XP-nSL values above 2 in a 10kb window were called significant (highlighted in gray). In addition, the variants with extremely high XP-nSL values above 4 are depicted in red. (C) The region of the genome under the prevalent additive QTL on the second chromosome, which also contains the most associated variants with the highest association scores, strong F_ST_ signal, and a significant signature of a partial sweep, harbors the *STAT-1* and *CG022363* genes (shown in red shaded area). The position of the associated variants is shown in red, the candidate gene in blue, and two neighboring genes in gray. For the depiction of all genes affected by associated mutations under the two QTLs see S12–S14 Figs. (D, E) The phylogenetic trees of the *STAT* and *CG022363* gene families were shown for *Caenorhabditis elegans*, *Drosophila melanogaster*, *Musca domestica*, *Anopheles gambiae*, *Mus musculus*, *Clunio marinus* candidate gene (blue), and *Clunio marinus* ortholog of the candidate gene (black). (F) *C*. *marinus* has two STAT genes: CG012971 (STAT-1) and CG022905 (STAT-2). The alignment, conserved domains, and percentage of conservation between the two amino acid sequences are shown.

Finally, although the multiple QTL mapping is crucial for investigating the most likely number of QTLs, it tends to overestimate the QTL intervals. Thus, to investigate the genes underlying the additive QTLs we relied on composite interval mapping conducted in 10cM windows (dark blue in Figs 2B, 2D, and 3A), while being informed by the QTL intervals resulting from the best binary phenotypic panels found by the EM algorithm (light green in Figs 2B, 2D, and 3A).

### Whole-genome sequencing reveals genetic variants associated with insensitivity to tidal turbulence in Ros-2FM

As discussed above, the resolution of the QTL mapping in our model system can theoretically go down to 2-3Mb which can still harbor several hundred genes. Therefore, in order to further identify specific genomic loci underlying QTL regions, we combined QTL mapping with genome-wide association analysis (Fig 3A and 3B). We performed whole genome sequencing of 20–24 field-caught males from nine *Clunio* populations differentially sensitive to tidal turbulence (Tables 1 and S1 and Figs 1A and S1) and called 746,887 SNPs and small indels. We used circular summary statistics (vector length) as the population-wide phenotypic score for sensitivity to tidal turbulence (Fig 3B and Table 1). We then applied the bayesian tool BayPass for calculating the strength of association of each of the variants to the insensitivity score (S5 Table) while using a kinship matrix to correct for population structure (S11 Fig). Out of 746,887 variants, 357 were significantly associated with sensitivity to tidal turbulence (Fig 3B, top panel). These variants affect 178 genes, as determined by SNPeff (S6 Table and S11 and S12 Figs). Most of the variants are located in non-coding regions and only a handful have potentially disruptive impacts on the respective genes (S11C and S11D Fig).

Crucially, the Ros-2NM population, which is sympatric to the insensitive Ros-2FM population, is sensitive to tidal turbulence (S1 Fig and Table 1). We can therefore ask if this phenotypic loss occurred as a result of a recent selective sweep due to local adaptation. To explore this, we first estimated genomic differentiation (F_ST_) between the two populations and found that the most prominent loci identified in the BayPass screen also have high F_ST_ values (Fig 3B–middle panel). Furthermore, if we assume that the causal genetic variant underwent positive selection as a result of a selective sweep, we can expect that it would leave a characteristic pattern of long high-frequency haplotypes and low genetic diversity in its vicinity [46]. The measure for a selective sweep occurring as a result of a local adaptation is calculated as the decay of haplotype homozygosity between the two populations [46] and is implemented in cross-population statistic XPnSL (nSL: number of segregating sites by length) [47] in selscan 2.0 [48] (for details see Methods section). The top 1% of the 10kb regions containing a cluster of alleles with high XPnSL values were considered to be candidate regions under selection in Ros-2FM (Fig 3B–bottom panel).

Finally, we combined the results from QTL analysis, the genome-wide association screen, and the selection screen. We identified the loci underlying additive QTLs (Figs 3C and S12) and found the orthologues in model organisms of all the genes in the vicinity of the associated mutations (Figs 3D, 3E, S13, and S14). The genomic region underlying the shared additive QTL on the second chromosome (Fig 3A) contains one cluster of variants with the highest association scores in BayPass, as well as high Fst and a selective sweep signature (Fig 3B). This cluster of variants affects three genes: signal transducer and activator of transcription 1 (*STAT-1*), *CG022363*, and *Lon* (Fig 3C). *CG022363* is an ortholog of the *Drosophila melanogaster CG32100* gene (Fig 3D), which plays a role in gravitaxis [49] but is otherwise poorly investigated. The STAT protein family is conserved in most vertebrates and invertebrates (Fig 3E). *Clunio*, unlike *Drosophila melanogaster*, has two paralogues: CG012971 (STAT-1) and CG022905 (STAT-2). STAT-1 is most likely the ancestral STAT protein: ortholog of *Anopheles gamibiae* STAT2 and *Mus musculus* STAT5a,5b, and 6; while STAT-2 is newly duplicated in *Clunio* (Fig 3E). The two *Clunio* STAT proteins are 83% identical in amino-acid sequence (Fig 3F). The most divergent protein domains in the two *Clunio* STAT proteins are the N-terminal domain, coiled-coil domain, and sh2 domain (Fig 3F). Lon is a highly conserved protease (S13F Fig) which is crucial for mitochondrial homeostasis [50].

As the QTL mapping explains at most 40% of the phenotypic variance, other loci of smaller effect must exist and are potentially picked up by the association analysis. We, therefore, explored all the genes identified by BayPass and SNPeff by conducting a gene ontology (GO) term enrichment analysis (S15 Fig). Out of 178 genes, 67 went into the GO analysis as they passed the criteria of having known orthologues, and 51 of those genes drove 78 significant GO terms (S15 Fig). Interestingly, gravitaxis was one of the highly significant GO terms. This result, together with the previous identification of the gravitaxis gene *CG022905* under the prevalent QTL, prompted us to look more closely into the genes with known roles in gravitaxis (S7 Table). We found that out of 27 such genes in Drosophila, 6 are on our list of genes potentially associated with the loss of sensitivity to tidal turbulence (S7 Table).

### Complex genetic basis for the loss of sensitivity to tidal turbulence in Jean-2NM population

**Fig 4:**
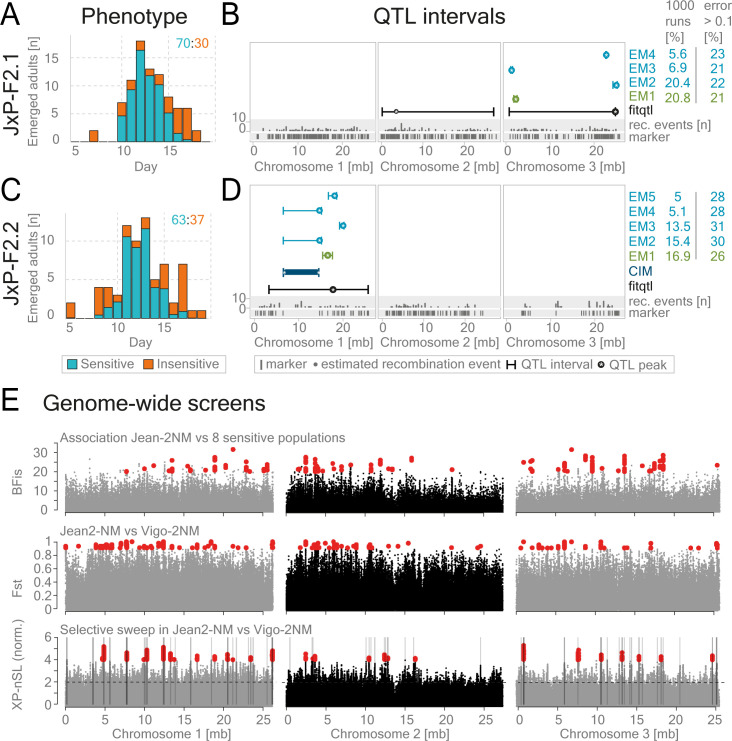
The oligogenic basis for the loss of sensitivity to tidal turbulence in the Jean-2NM population. QTL mapping in two Jean-2NM x Por-1SL intercross families was performed to find genomic regions harboring genes responsible for the loss of mechanical entrainment in Jean-2NM (A-D). (A, C) The proportion of insensitive (orange) and sensitive (blue) individuals found on each day was calculated based on estimated probabilities (S16 Fig). The ratio of the two phenotypes is indicated in the top right corner. (B, D) QTL intervals are given for: composite interval mapping–dark blue, fitqtl: additive loci–black, EM-algorithm–light blue. The green marks the phenotypic panel with the highest convergence in EM analysis, and the lowest error (see methods QTL mapping/EM-pipeline. Raw data and full statistics are given in S8 Table). (E) Association analysis was performed to find mutations associated with the loss of sensitivity to tidal turbulence in the Jean-2NM population. (E-top): We screened for variants associated with the loss of sensitivity to tidal turbulence using 769.379 variants called from 210 individuals belonging to 9 differentially sensitive populations (S1 and S19 Figs). Bayesian factor (BFis) is plotted for each variant along the three chromosomes. We found 173 significantly associated SNPs and indels (BFis > 20, eBPis > 2, XtXst > 20.02; see Methods section for details) marked in red. The list of SNP effects and genes affected by them is given in S9 Table. Raw data are published on Max Planck Repository Edmond https://doi.org/10.17617/3.HUYLPR). (E-middle and bottom) To find loci under selection in Jean-2NM that could be responsible for the loss of sensitivity, we contrasted it with the closest turbulence-sensitive population Vigo-2NM. (E-middle) plots show the results of genomic differentiation analysis (Fst) between Vigo-2NM and Jean-2NM. Red marks Fst values above 0.5. (E-bottom) Plots depict the results of the selective sweep analysis in Jean-2NM as compared to the Vigo-2NM. The top 1% 10kb regions under selection are gray.

As detailed above, complementation crosses between the two insensitive strains identified a separate origin for the insensitivity to tidal turbulence in Jean-2NM and Ros-2FM. To corroborate this finding, we further explored the genetic basis of insensitivity in the Jean-2NM population (Fig 4).

QTL mapping was conducted in two intercross families: in the JxP-F2.2.3 family we found one additive QTL on the first chromosome, while in the JxP-F2.1.6 family two additive QTLs on chromosomes 2 and 3 appeared (Figs 4A–4D, S16, and S17 and S8 Table). Interestingly, while the ratio of sensitive to insensitive individuals was consistently 73:27 in three independent intercross families including JxP-F2.1 (S18 Fig), the JxP-F2.2 family had a unique ratio of 62:38 (S18 Fig). This could indicate that the genetic basis for the insensitivity in JxP-F2.2 is unique and may explain why we found different QTLs in JxP-F2.1.6 and JxP-F2.2.3. In addition, this finding suggests that there is an oligogenic origin for the trait and that the alleles responsible for the loss of sensitivity are not fixed in the Jean-2NM population.

We then performed the same genetic screens in Jean-2NM as in Ros-2FM (Fig 4E). The association analysis identified 173 SNPs significantly associated with the phenotypic loss in Jean-2NM as compared to the eight sensitive populations (Fig 4E–top panel and S9 Table). As in the Ros-2FM association analysis, most associated SNPs are found in the non-coding regions of the genome (S19 Fig). To investigate potential selective sweeps in Jean-2NM, we tried contrasting it with the closest turbulence-sensitive population we had: Vigo-2NM (Figs 4E and S1). However, since the two populations are geographically quite far from each other, the F_ST_ values were very high overall (Fig 4E–middle panel). Thus, Vigo-2NM is not the most suitable reference population for discovering reliable selective sweeps in Jean-2NM (Fig 4E–bottom panel). Taken together, due to the complexity of the QTL mapping results in Jean-2NM, as well as the lack of prominent peaks in the association analysis, we were not able to identify candidate genes with enough precision. Nevertheless, the absence of a prominent QTL on chromosome 2 in Jean-2NM corroborates the finding that this phenotype was lost independently in Ros-2FM and Jean-2NM.

## Discussion

### Loss of sensitivity to mechanosensory entrainment: result of convergent evolution?

Loss-of-function alleles were once only associated with deleterious mutations, and loss of genes with the loss of redundant gene duplications. It is now understood that the loss of alleles and genes can drive adaptive phenotypic diversity [39,40]. Furthermore, in contrast to the early evolutionary theories, we now come to understand that convergent evolution is more of a rule than an exception. A few recent studies show that the loss of traits can happen as a result of convergent evolution: repeated eye loss in Mexican cavefish [51] and the loss of flight in paleognathous birds [52].

*Clunio* colonized the European Atlantic coast from south to north following the last ice age about 10.000 to 20.000 years ago [36], which in evolutionary time is rather short. Given that insensitive populations are found in the south (Fig 1A), one could assume that insensitivity is the ancestral state. However, only two out of ten tested populations are insensitive to tidal turbulence, and the most southern population, Vigo-2NM, is sensitive to this cue (Figs 1A and S1). Our data also show that insensitivity has at least two different recessive genetic bases. The obvious difference in Ros-2FM and Jean-2NM in identified QTLs (Figs 2 and 4A–4D) and the positions of the associated SNPs (Figs 3 and 4E), corroborates the results from the complementation cross (Fig 1C) and leads to the conclusion that this trait indeed evolved independently in the two locations. Furthermore, sensitivity presumably involves a complex signalling pathway that is more difficult to evolve several times from insensitivity, than to "break it" with a few mutations. Taken together, this hints that the ancestral *Clunio* population was likely sensitive, and the insensitivity in certain populations may be considered a loss of trait. The adaptive value of this loss remains speculative (see below). But if this phenotype has adaptive value, we may have uncovered an example of recent convergent evolution in the process of local adaptation to different timing habitats.

### Evolution of differential sensitivity to circalunar synchronizers

In agreement with Neumann [38], the northern populations (Por-1SL, Bria-1SL, He-1SL, and Ber-1SL) are very sensitive to tidal cues while southern ones are less sensitive or entirely insensitive (Jean-2NM, Plou-2NM, Ros-2NM, Ros-2FM, and Lou-2NM). He argued that moonlight is an ill-suited zeitgeber in the north due to the low position of the moon on the horizon [38]. However, that does not explain why tidal turbulence would be unreliable in the south. There is no obvious advantage for Ros-2FM or Jean-2NM to lose the sensitivity to this cue since the tides are as strong and predictable in those locations as in any other tested location.

We also observed that populations most sensitive to the tidal turbulence have a semi-lunar period in adult emergence, i.e. they emerge twice a lunar month, while less sensitive populations have a lunar period (except Vigo-2NM). Tidal turbulence is a semi-lunar zeitgeber as it comes from the tides, and it could therefore be a more appropriate cue for the populations emerging twice a month in contrast to those that are emerging once a month for which moonlight, as a monthly zeitgeber, might be a more suitable cue.

Furthermore, we discovered that the two sympatric populations in Roscoff are differentially sensitive: Ros-2NM is sensitive to tidal turbulence, and Ros-2FM is insensitive to tidal turbulence (S1M and S1O Fig). Although we tested the two zeitgebers moonlight and tidal turbulence separately in the laboratory, they are perceived together in the wild. Furthermore, as the timing of the tides changes along the Atlantic coast, their phase-relationship varies in different habitats [37]. Thus, if the two zeitgebers set the phase differently, losing sensitivity to one of them can be an evolutionary strategy to set the phase according to the most informative zeitgeber. In line with that, we find the same QTL locus harboring STAT-1 and CG022363 as one of the QTLs responsible for the phase-difference between Ros-2FM and Ros-2NM [53].

### Genes responsible for the loss of sensitivity to tidal turbulence in Ros-2FM

Tidal turbulence is a vibration, perceived by the mechanosensory nervous system, and mechanosensory pathways are even in model organisms still largely unknown. Most molecular players were identified in genetic screens on phenotypes associated with defects in mechanosensory systems in *Drosophila melanogaster*, *Caenorhabditis elegans*, and *Mus musculus* [54]. Genes found in these analyses are not only directly involved in mechanosensation. They include ion channels, the tethering of the ion channels, extracellular matrix and cytoskeleton; but also indirectly the development of the sensory organs or the function and development of the cells downstream in the neuronal circuits [54]. Many complex phenotypes are polygenic in origin, which makes simple gene–function relationships hard to infer. Additionally, mutations in regulatory regions, rather than mutations in coding regions, are found to shape most emerging phenotypes [52]. Similarly, the loss of complex phenotypes has been shown to be driven by divergence in cis-modulatory elements of developmental genes in the loss of limbs in snakes and degeneration of eyes in subterranean mammals [55]. Interestingly, here we also found that the majority of potentially causal SNPs hit intergenic regions (S11C Fig and S6 Table), suggesting they are rather affecting regulatory elements. Therefore, we investigated both, the region of the genome with the highest association score (Fig 3C), but also other potential candidate genes that showed up in the association analysis with BayPass (S12–S15 Figs and S6 and S7 Tables), irrespective of whether there was a signature of selection at these loci.

### Gravitaxis: Potential role of chordotonal organs

We found *CG022363* to fall into the region with the highest association score (Fig 3C). This gene is an ortholog of the *Drosophila CG32100* gene, which has a role in gravitaxis although the exact molecular function of this gene remains unknown [49]. Graviception is a function of the mechanosensory system, and as is the case with all mechanosensory functions, it is poorly understood on a molecular level. To this day, most of the molecular machinery was identified through genetic screens of behaviors associated with impaired gravitaxis [49]. As a result, 27 genes were associated with gravity-sensing in *Drosophila*. Some detect gravity directly, namely *inactive*, *nanchung*, *painless*, and *pyrexia* [56]. But the majority seem to have an indirect role most likely in the development of the sensory organs: *alan shepard*, *escargot*, *broad*, *cryptochrome*, *nemo* and others [49,56]. Strikingly, out of those 27 genes, we found 6 that were associated with loss of sensitivity to tidal turbulence in Ros-2FM (S7 Table): *shep*, *snaill1_CG000103*, *broad*, *cryptochrome1*, *nemo*, and the above-mentioned *CG022363*. In line with that, gravitaxis was found as one of the top GO terms (S15 Fig). Three of the six belong to the 15 candidate genes under the QTL regions: *shep*, *snaill1_CG000103*, and *CG022363* (S12 Fig). In *Drosophila shep* is involved in neuronal development and remodeling of the sensory neurons [57,58] and *escargot* has a role in neurogenesis [59]. Therefore, it is likely that they are indirectly involved in gravitaxis in *Drosophila* by contributing to the development of the sensory organs responsible for detecting gravity. *Drosophila* larvae detect both vibration and gravity via chordotonal organs [60,61]. In addition, chordotonal organs are necessary for the mechanosensory entrainment of the circadian clock in *Drosophila* adults [14]. Taken together, it is possible that chordotonal organs are responsible for mechanosensory entrainment of the circalunar clock in *Clunio* as well. Mutations in genes responsible for the development of the chordotonal organs could lead to impaired gravity sensing as well as detection of vibration and thus impair mechanosensory entrainment of the circalunar clock in Ros-2FM.

### STAT-1 locus

Together with *CG022363*, *STAT1* falls into the region with the highest association score (Fig 3C). Signal Transducer and Activator of Transcription (STAT) protein is a part of the evolutionary conserved JAK-STAT pathway that controls developmental decisions and participates in the immune response [62]. Archetypical members of each of the components were present at the time of the emergence of Bilateria: JAK, STAT, SHP, and the three SOCS proteins [63]. STAT proteins were duplicated many times throughout metazoan evolution, and while some pseudogenized, many evolved into novel genes through rapid sequence diversification and neofunctionalization [62]. Insect STATs form a single clade in phylogenetic analyses and constitute an ancient class of STATs together with mammalian STAT5 and 6 [62]. While most insect species like *Drosophila melanogaster* and *Apis mellifera* have a single STAT whose function remains conserved [62], in others like *Anopheles gambiae* STAT duplicated and the new gene acquired diverse functions. Duplicated *Anopheles* STAT has a role in defense against bacteria [64], *Plasmodium* infection [65], and innate immunity [66]. In addition, duplicated STAT acts as an upstream regulator of the evolutionarily conserved STAT protein [65].

In contrast, in vertebrates, all components of the JAK-STAT pathway duplicated several times and STAT proteins attained specialized functions in various cells. Interestingly, the expression of the TrpA1 mechanosensitive channel is regulated via the JAK-STAT pathway in nociceptive neurons in mice [67]. Similarly, STAT3 is necessary for the differentiation and regeneration of inner ear hair cells, the basic mechanosensory receptors for hearing and balance in zebrafish [68]. Finally, the JAK-STAT pathway is directly coupled to the mechano-gated channels in various non-neuronal cells, regulating gene expression downstream of the channel activation [69–74].

*Clunio marinus* has two STAT proteins: CG012971 (STAT-1), the ortholog of *Anopheles gamibiae* STAT2, and *Mouse* STAT5a,5b and 6; and CG022905 (STAT-2) (Fig 3D). Two *Clunio* STATs are 83% identical in amino-acid sequence (Fig 3E), while *Anopheles* STATs share only 47% overall sequence identity [62]. Two *Clunio* STATs differ the most in the N-terminal domain which has a role in nuclear translocation and protein-protein interactions, and the coiled domain which is involved in nuclear export and regulation of tyrosine phosphorylation [63]. This indicates that the two STATs could be regulated differently or be a part of different signalling pathways by interacting with different proteins and thus obtaining different roles.

Taken together, we can speculate that *Clunio* STAT-1 has a role in the perception of tidal turbulence by being involved in the development or differentiation of mechanosensory organs, or mechanosensory receptors appropriated this JAK-STAT pathway for regulation of gene expression. Further functional analysis is necessary to test this hypothesis. If proven, this would be the first evidence of a STAT role in mechanosensation in invertebrates.

### Modulation of the circadian clock is responsible for the lack of mechanosensory entrainment of the circalunar clock?

The mechanosensory cue comes from the tides, and as such it consists of a 6h 10min stimulus followed by a 6h 15min break. Its onset shifts every day by 50min. As a consequence, the tidal pattern of mechanical disturbance occurs at the same time of day every 15 days, i.e. after half a lunar cycle. Thus, lunar phase can be detected by setting a daily window of mechanoreceptor sensitivity [38], which will only overlap with the presence of mechanical cues during specific lunar phases. Such a daily sensitivity window would be governed by a circadian clock. Interestingly, we found several circadian clock genes in our genome-wide screen: *cry1*, *per* and *clk* (S6 Table). It is tempting to speculate that modulation in the circadian clock might have an effect on the lack of mechanosensory entrainment in the Ros-2FM population. In this scenario, loss of mechanosensory entrainment would not be due to a malfunction in the sensory pathways, but due to a misinterpretation of the cue in a circadian context.

Here we show for the first time a convergent loss of sensitivity to tidal turbulence in two *Clunio* populations. We found several loci to be responsible for this loss. A detailed analysis suggests that in one of the populations the JAK/STAT pathway and gravitaxis may play a prominent role in the detection of tidal turbulence. While in Baltic and Northern European populations complete lunar arrhythmicity seems to be a highly polygenic trait [75], the selective loss of sensitivity to a zeitgeber seems to have a less complex, oligogenic basis. If in the future tools for molecular manipulation of *Clunio* are developed, this setting is a good starting point to identify novel genes and pathways involved in mechanosensation.

## Methods

### *Clunio* cultures

*C*. *marinus* cultures were established from different locations (S1 Table) and maintained in the laboratory according to Neumann [27]. Around 1000 larvae were kept in 20x20x5 cm plastic boxes with sand from the natural habitat and 15‰ seawater. They were fed twice a week with diatoms (Phaeodactylum tricornutum, strain UTEX 646). Nettle powder was added twice a month with each water exchange. *Clunio* larvae were raised under a 16h light and 8h darkness regime and a temperature of 18°C. In experiments with moonlight entrainment, the artificial moonlight was simulated with neutral white LED ~4000K light (Hera 610 014 911 01) on 4 consecutive nights every 30 days. The 24-hour period when moonlight was first applied was marked as day 1. In the experiments with mechanosensory entrainment, cycles of vibration were used to simulate tidal turbulence in a setup established by Neumann [31]. Briefly, an electromotor generating vibration of 50 Hz, 30dBa above background noise was attached to the shelves with *Clunio* cultures and controlled by a custom-made “tidal clock”. The clock kept the motor on for 6h 10 min and off for 6h 15min which gave a 12.4-hour tidal rhythm. The onset of vibration shifted every day by 50 min which resulted in a 144-day semi-lunar cycle. The day when vibration started in the middle of their subjective night was arbitrarily marked as day 1. Phenotypes were recorded by collecting emerged adults from three culture boxes per strain every day for at least 60 days or two lunar cycles.

### Crossing experiments

To explore the genetic basis of insensitivity to tidal turbulence, we crossed the insensitive Ros-2FM or Jean-2NM strains with the sensitive Por-1SL strain, as well as the two insensitive strains with each other (S2 Table). Detailed description can be found in the S1 Methods.

### QTL mapping

QTL mapping was performed to identify genetic regions harboring genes where natural variants that underlie the loss of sensitivity to tidal turbulence are segregating. Two families from the Ros-2FM x Por-1SL cross were chosen for QTL mapping: (Ros-2FMxPor-1SL)x(Ros-2FMxPor-1SL)-F2.1 in the further text referred to as RxP-F2.1, and (Ros-2FMxPor-1SL)xRos-2FM-BC.1 in the further text referred to as RPxR-BC.1 (S2 Table). Similarly, two intercrosses of Jean-2NM x Por-1SL families were selected: (Jean-2NMxPor-1SL)x(Jean-2NMxPor-1SL)-F2.1.6 and (Jean-2NMxPor-1SL)x(Jean-2NMxPor-1SL)-F2.2.3in the further text referred to as JxP-F2.1.6 and JxP-F2.2.3.

### Phenotyping (S3 Fig)

Emergence data was collected for parental, F1 and F2, and BC generations, and lunar emergence days under turbulence entrainment were assigned as described above (S1 Script and Table 1 and S1 and S2 Figs). To resolve the problem of the overlapping “sensitive” and “insensitive” phenotypes emerging during the peak in the F2 and BC progeny, we designed a pipeline to calculate the probability of finding “sensitive” and “insensitive” individuals on each day. For more details see S1 Methods.

### Genotyping

DNA was extracted from adults collected in crossing experiments with the salting-out method [76], it was aplified using RepliG, and single-digest or double-digest RAD sequencing was performed [77–79]. A detailed protocol can be found in the S1 Methods. The script containing read processing, mapping, genotype calling and filtering for informative variants is given in S2 Script.

### Read processing and mapping

For discriminating individuals, P1 and P2 adaptors contained unique barcode sequences (S10 Table). Raw reads were trimmed to remove adapters and low-quality bases with Trimmomatic v0.38 [80]. Trimmomatic parameters used for paired-end reads were ILLUMINACLIP:<PE_adapter_file>:2:30:10:2:true LEADING:20 TRAILING:20 MINLEN:50 and for single-end reads ILLUMINACLIP:<SE_adapter_file>:2:30:10 LEADING:20 TRAILING:20 MINLEN:50. For paired-end library RxP-F2.1, overlapping read pairs were assembled into single reads with PEAR v.0.9.10 [81] using default parameters. Paired (PEAR unassembled) and single reads (PEAR assembled and unpaired reads from R1 or R2 after adapter trimming) were mapped independently with NextGenMap v0.5.5 [82] to the CLUMA2.0 reference genome (available at https://doi.org/10.17617/3.42NMN2) with default parameters except for—min-identity 0.9 and—min-residues 0.9. Read groups were specified during mapping using—rg-id and—rg-sm. The independently mapped reads were then merged into a single file using samtools v1.9 [83] merge, with parameters -u -c -p. For single-end libraries, trimmed reads were directly mapped with NextGenMap with previously mentioned parameters. Mapped reads were sorted and indexed with samtools sort and samtools index respectively.

### Variant calling

Single nucleotide polymorphisms (SNPs) and insertion-deletion (indel) genotypes were called using GATK v3.7-0-gcfedb67 [84]. Steps include initial genotype calling using GATK HaplotypeCaller with parameters—emitRefConfidence GVCF and -stand_call_conf 30, filtering of variants using GATK SelectVariants with ’-select DP > 30.0’, recalibration of base qualities using GATK BaseRecalibrator with ‘-knownSites’, preparing recalibrated BAM files with GATK PrintReads using -BQSR and finally, recalling of genotypes using GATK HaplotypeCaller with previously mentioned parameters. Individual VCF files were combined into a single file using GATK GenotypeGVCFs.

### Informative variants and genotype matrix

VCF files were filtered for minimum genotype quality (minGQ) 20, minor allele frequency (maf) 0.10, and maximum fraction of samples having missing genotypes (max-missing) 0.60. Genotypes were coded as ‘AA’, ‘AB’, or ‘BB’ based on the inferred inheritance pattern (S11 Table). To maximize the number of informative markers in a backcross, we included markers for which both parents were heterozygous or, the F1 parent was heterozygous and the Ros-2FM parent was homozygous (S11 Table). To infer from which parent the ‘A’ or ‘B’ allele comes from at ambiguous loci, we chose genotypes based on the consistency of the genotypes along the chromosome (i.e. the assignment that had a smaller number of genotype switches across the individuals in the BC progeny) (S11 Table, consistency genotype assignment in S2 Script). The final genotype matrix was manually inspected for each mapping family, before importing it into R/qtl with *read*.*cross* function, within which the flag *map*.*function = "kosambi"* was used to generate the linkage map. We used „*kosambi*”function as it does not assume that the markers are normally distributed. Marker order and genotype errors were further investigated in R/qtl following the best practices suggested by the authors [85]. One inversion was identified in the right arm of the second chromosome in the RxP-F2.1 family and the order of markers was inverted in that region. The final number of markers was 117 for RPxR-BC.1 and 137 for the RxP-F2.1 family. The final genotype matrixes are given in S3 Table.

Samples from parents’ and F1s of the two Jean-2NMxPor-1SL families, unfortunately, had very few good genotypes. Thus, we designed an alternative approach for reconstructing the recombination matrix. For details see S1 Methods.

### QTL mapping

Standard interval mapping and multiple QTL mapping were done with R/qtl package functions: *scanone*, *scantwo*, and *fitqtl* [85]. QTL intervals were estimated with *bayesint* function. Composite interval mapping was analyzed in Windows QTL Cartographer Version 2.5_011 (number of covariates 5, window 10 cM) [86]. In addition, to confirm the model found by *fitqtl* multiple QTL mapping, we used the Bayesian QTL mapping R package “qtlbim” [87]. Function *qb*.*best* was used to identify the best model, and *qb*.*scanone* to compare additive and epistatic QTLs found by R/qtl.

### *Expectation-maximization (EM) algorithm* (S5 Fig)

To explore the effect of uncertainty in phenotyping on the QTL mapping results, we devised an EM algorithm to assign binary phenotypes to the entire dataset (S5 Fig). For details see S1 Methods. The script can be found in S3 Script.

### Association analysis

The complementation cross indicated that the genetic basis for the loss of sensitivity is different in the two populations (Fig 1). Therefore, the two insensitive populations were analyzed separately. To identify variants associated with the loss of sensitivity to tidal turbulence in Ros-2FM we performed a genome screen on 746.887 SNPs and small indels called in 210 males from nine populations differentially sensitive to tidal turbulence (S4 Table). Similarly, to find potentially causative mutations in Jean-2NM, we used a dataset of 769.379 SNPs and indels from 210 males from Jean-2NM and the same eight populations sensitive to tidal turbulence.

### Genotyping

DNA from field-caught males stored in 100% ethanol was extracted using the salting-out method [76]. Genomic DNA was amplified with standard RepliG protocol (REPLI-g Mini Kit QIAgen 150025). Whole genomes of 20–24 adults from nine populations were individually sequenced on Illumina HiSeq3000 with paired-end 150-bp reads and average coverage of 20x (S4 Table).

### Read processing

Reads from several sequencing runs were merged with the *cat* function. Adapters were trimmed using Trimmomatic tool [80] and the following parameters: ILLUMINACLIP <Adapter file>: 2:30:10:8:true, LEADING:20, TRAILING:20, MINLEN:75. Overlapping read pairs were assembled using PEAR with the following parameters: -n 75 -c 20 -k [81]. Reads were mapped using bwa mem version0.7.15-r1140 [88] using the latest Cluma_2.0 reference genome (available at https://doi.org/10.17617/3.42NMN2). The independently mapped reads were then merged into a single file, filtered for -q 20, and sorted using samtools v1.9 [83].

### Variant calling

SNPs and small indels were called using GATK v3.7-0-gcfedb67 [84]. All reads in the q20 sorted file were assigned to a single new read-group with the ‘AddOrReplaceReadGroups’ script with LB = whatever PL = illumina PU = whatever parameters. Genotype calling was then performed with HaplotypeCaller and parameters—emitRefConfidence GVCF -stand_call_conf 30, recalibration of base qualities using GATK BaseRecalibrator with ‘-knownSites’. Preparing recalibrated BAM files with GATK PrintReads using -BQSR. Recalling of genotypes using GATK HaplotypeCaller with previously mentioned parameters. Individual VCF files were combined into a single file using GATK GenotypeGVCFs.

### BayPass genotype matrix

The genotype matrix for BayPass association analysis [89] was generated by filtering for minor allele frequencies larger than 0.05, the maximal number of missing values per variant was set to 20%, the maximal number of alleles was 2, and minimal read quality minQ was set to 20 with VCFtools (0.1.14) [90]. Allele count per population was calculated using the VcfR package [91]. Briefly, a previously filtered vcf table containing 24 individuals from 9 populations was separated into vcf files containing individuals from distinct populations. Individual vcf files were read with read.vcfR function and allele frequency per population per site was calculated using the gt.to.popsum function. Population allele frequencies were then combined into a genotype matrix.

### Phenotyping

Sensitivity to turbulence was estimated for each population using summary circular statistics (see methods section QTL mapping/Phenotyping). Vector length was used as a phenotypic score standardized using -scalecov flag so it had a mean of 0 and a standard deviation of 1, (S5 Table).

### BayPass

BayPass was run with 3 random seeds (1, 1988, 11273), and the median of BFis, eBPis, XtXst, and -log10 p-value of XtX was calculated. To find the correct significance threshold for XtX statistics, pseudo-observed data set (POD) was generated by sampling 100.000 SNPs with R function *simulate*.*baypass* and found that 1% of XtXst POD values was 21.67 in Ros-2FM dataset, and 20.02 in Jean-2NM. To subset highly associated variants in Ros-2FM, we filtered for BFis > = 20, eBPis > = 2 and XtXst > = 21.67 (S11A Fig) and BFis > = 20, eBPis > = 2 and XtXst > = 20.02 in Jean-2NM (S19C Fig). Association analysis in BayPass is corrected for the population structure based on a kinship matrix Ω.

### SNPeff

SNP effects were analyzed in CLUMA2.0_M, a version of the reference genome that contains manual curations to the reference sequence made during genome annotation (available at https://doi.org/10.17617/3.42NMN2). SNPs were transferred from CLUMA2.0 to CLUMA2.0_M using a Python3 script (S4 Script), which creates a map of positions from CLUMA2.0 to CLUMA2.0_M by accounting for insertions and deletions. As input, the script uses a GFF file with manual reference edits, exported from Web Apollo version 2.5.0 [92]. With the CLUMA2.0_M reference sequence, the location and putative effects of the SNPs and indels relative to CLUMA2.0_M gene models were annotated using SnpEff 4.5 (build 2020-04- 15 22:26, non-default parameter `-ud 0’) [93]. The complete list with the number of variants with distinct effects is given in S6 and S9 Tables.

### Phylogenetic trees

The identity of the 15 candidate genes was explored by the reciprocal blast between *Clunio* and *Drosophila melanogaster* protein sequences. eggNOG 5.0 database was then used to identify orthologs in other model organisms: *Anopheles gambiae*, *Mus musculus*, *Homo sapiens*, and *Caenorhabditis elegans* [94]. The most distant protein sequence in eggNOG phylogenetic trees was taken as an outgroup sequence. Protein sequences were then aligned, and phylogenetic trees were created in QIAGEN CLC Main Workbench version 7.9.3. Bootstrap values in 1000 runs were reported (Figs 3D, 3E, and S13–S14).

### Selective sweep analysis

To investigate if the associated loci evolved as a result of a recent selective sweep in the process of local adaptation, we calculated cross-population nSL (number of segregating sites by length) developed by [95]. XP-nSL is designed to detect selective sweeps due to local adaptation within a query population by comparing its integrated haplotype homozygosity (iHH) with one of a reference population. Here, positive scores suggest long haplotypes in population A with respect to population B and a potential sweep in A, whereas negative scores suggest long haplotypes in B with respect to A. nSL, in contrast to EHH, was developed to accommodate the lack of genetic maps in favor of physical maps [47]. We used selscan 2.0 as it was recently revised to work with unphased multi-locus genotypes [48,95]. Details can be found in the S1 Methods.

### Genetic differentiation (fst)

To provide a bridge between the association analysis conducted on ten populations, and the cross-population selective sweep analysis calculated between the two populations (see Methods section on association analysis and selective sweeps), we estimated genetic differentiation between those two contrasted populations: Ros-2FM compared to Ros-2NM and Jean-2NM compared to Vigo-2NM. The same vcf files containing GATK-called SNPs and indels used for selective sweep analysis were used (see Methods section on selective sweeps). Genetic differentiation between the two populations (fst) was estimated using vcftools version 0.1.14 [90] parameters—weir-fst-pop—fst-window-size 1—fst-window-step 1.

### GO term enrichment

To investigate if the genes identified by BayPass and SNPeff perform some of the known biological functions, we ran Gene Ontology (GO) term enrichment. We previously annotated 5,393 out of 15,193 *C*. *marinus* genes with GO terms [96]. In our current reference genome CLUMA2.0, 5436 out of 13751 genes were annotated with GO terms. In brief, GO terms were annotated using the longest protein sequence per gene with mapper-2.0.1.[97] from the eggNOG 5.0 database [94], using DIAMOND [98], BLASTP e-value <1e-10, and subject-query alignment coverage of >60%. Only GO terms with “non-electronic” GO evidence from best-hit orthologs restricted to automatically adjusted per-query taxonomic scope were used. To assess the enrichment of “Biological Process” GO terms, the weight01 Fisher’s exact test was implemented in topGO (version 2.42.0, R version 4.0.3) [99].

## Supporting information

S1 Fig **Clunio populations are differentially sensitive to moonlight or tidal turbulence** (A) Origin of the *Clunio* strains. Strains are color-coded and their names are depicted in the body of the graph. (B-U) Graphs show the fraction of emerged individuals entrained under laboratory conditions by either artificial moonlight (four nights of light every 30 days) or tidal turbulence (vibration of ~50 Hz 30dB above background noise in 6h 10min ON– 6h 15min OFF intervals resulting in a 15-day pattern). The total number of individuals, exact names of geographical locations, and the year when strains were established are given in S1 Table. Strains differ in the period, phase of emergence, and sensitivity to the synchronizers. The emergence of strains considered insensitive to moonlight or tidal turbulence is marked in black. Map data was obtained from https://www.naturalearthdata.com/downloads/50m-physical-vectors/.
(PDF)

S2 Fig Sensitivity to tidal turbulence is genetically determined and a dominant trait. Crossing experiments were performed to assess the inheritance of sensitivity to tidal turbulence. Graphs show fractions of emerged adults per day in parental populations, F1 and F2 (F1xF1) generations. (A) Intercross between Por-1SL and Ros-2FM. (B) Intercross between Por-1SL and Jean-2NM. The total number of individuals per generation is listed in S2 Table. The color of the bars represents increasing levels of sensitivity to tidal turbulence from sensitive (light gray) to insensitive (black).
(PDF)

S3 Fig Schematic overview of the QTL mapping strategy. Left: Phenotyping strategy. Light and vibration were logged throughout the experiment and used to calculate the first day of the entrainment as the day when vibration starts in the middle of their subjective night. Emerged adults were collected and the number of emerged individuals per day was recorded. Circular summary statistics were used to test if there was a phase-shift in emergence rhythms between parental, F1, F2, and BC generations. If there was a phase shift, emergence days were corrected so that the only phenotype assessed is rhythmicity. To generalize the emergence distributions, kernel density estimates were calculated (bandwidth = 10) for each generation. The probability of finding sensitive and insensitive individuals on each experimental day in F2 or BC progenies was calculated according to the given equations. The probability of finding an insensitive individual was used as a phenotypic score. In addition, a reduced dataset was generated by removing individuals with uncertain phenotypes between 0.3 and 0.7. Remaining individuals with probability phenotypes > 0.7 or < 0.3 were given binary phenotypes 1 and 0 respectively. Right: QTL mapping strategy. Several mapping pipelines were tested to examine additive and epistatic QTLs. Full and reduced datasets of each crossing family were analyzed using interval mapping (scanone and scantwo), composite interval mapping (WinQTL cartographer), and multiple QTL mapping (fitqtl and qtlbim). See methods section QTL mapping.
(PDF)

S4 Fig Calculating probability phenotypes for RxP-F2.1 and RPxR-BC.1 family. (A-F) The fraction of emerged adults per generation is shown on a circular plot together with the mean and median vectors. (A) Por-1SL strain. (B) Ros-2FM strain. (C) RxP-F1.1 and RxP-F2.1 generation. (D) RxP-F1.2 three crossing families were raised together (gave rise to RPxR-BC.1). (E) RxP-F2.1 is a F1-24 x F1-24 intercross. (F) RPxR-BC.1 is a backcross of an F1.2 individual to Ros-2FM. (G-H) Kernel density estimates for parental, F1, and F2/BC generations for each of the two mapping families. RxP-F2.1 crossing family shows a 2-day phase shift as compared to Por-1SL. RPxR-BC.1 crossing family does not show considerable phase-shift. (I-J) Bar graphs show probabilities of finding sensitive Por-1SL-like (white) or insensitive Ros-2FM-like (black) individuals on each day in the two crossing families RxP-F2.1 (I) and RPxR-BC.1 (J).
(PDF)

S5 Fig An expectation-maximization (EM) algorithm. An EM algorithm was designed to generate optimized binary phenotype panels to a crossing family given the calculated probability of finding insensitive individuals on each experimental day. For a detailed explanation see the S1 Methods/QTL mapping/EM Expectation-maximization (EM) algorithm paragraph.
(PDF)

S6 Fig Ros-2FMxPor-1SL QTL mapping full analysis. Complete QTL mapping results for the two crossing families (RxP-F2.1 and RPxR-BC.1) and two datasets each (F = full and R = reduced) are given. (A-D) Bar graphs show the number of emerged individuals per day. The predicted ratio of insensitive (black) and sensitive (white) individuals is plotted. (E-H) LOD scores of interval mapping analysis (*scanone*) are designed to detect additive QTLs. Probability phenotypes were used for full datasets and binary phenotypes for reduced datasets. The significance threshold (dashed line) was estimated in 1000 permutations with a 5% cutoff. (I-L) CIM analysis with backward regression method, 5 control markers, and a window size of 10 cM. Threshold values are given in S3 Table. (M-P): LOD scores of scanone on EM-optimized binary phenotypes. Results are shown for panels obtained in at least 5% of the cases in 1000 runs (S3 Table). The significance threshold (dashed line) was estimated in 1000 permutations with a 5% cutoff. (Q-T) LOD scores of significant QTLs in multiple QTL mapping pipeline (*fitqtl*). Black lines: additive QTLs, gray lines: QTLs in epistasis. p-value of F statistic is marked: * p-value < 0.05; ** p-value of <0.01. *Fitqlt* statistics are given in S3 Table. (U-AB) To find the best model for multiple QTL mapping (*fitqtl*) we also tried the *qtlbim* package. (U-X) We first ran LPD (Log Posterior Density) scan that uses Bayesian model averaging to explore the most probable models. Most likely additive QTLs are shown in black and most likely QTLs in epistasis are shown in gray. (Y-AB) The “*best*” function of the *qtlbim* package then selects the most probable model. The larger the font size the larger posterior probability the pattern has. The 2-D multidimensional scaling (MDS) projection is based on the square of the attenuation. If the loci agree exactly, there is no attenuation. The best model is marked in red. The numbers represent chromosomes. (U) The best model for the RPxR-BC.1 full dataset contains 3 QTLs and one epistatic interaction on chromosomes 1, 2, 3, and 1:3. (V) The best model for the RPxR-BC.1 reduced dataset is 1, 2. (W) The best model for the RxP-F2.1 full dataset is 2, 3. (X) The best model for RxP-F2.1 reduced dataset is 1, 2.
(PDF)

S7 Fig Ros-2FMxPor-1SL QTL mapping: epistatic interactions. To scan for QTLs in epistasis, we used the *scantwo* function (*rqtl* package). Datasets are labeled (left gray; F = Full and R = Reduced) and correspond to S6 and S8 Figs. (A, C, E, G): *Scantwo* heatmaps for three chromosomes show LODf in the lower right corner that measures the improvement in the fit of the full two-locus model over the null model and indicates the evidence for at least one QTL with allowance for interaction. LODi heatmap is plotted in the upper left corner and measures the improvement in the fit of the full model over that of the additive model, and so indicates evidence for an interaction. Significant QTL epistatic interaction is marked by a red circle. (B, D, F): The epistatic effect for each of the significant interactions on the left is shown. The marker that shows the strongest interaction on each chromosome was selected and its location is marked in gray letters.
(PDF)

S8 Fig Ros-2FMxPor-1SL QTL mapping: intervals and phenotype scores. Plotted are QTL intervals and phenotype panels for the QTL analysis (S6 Fig). (A, C) QTL intervals: composite interval mapping–orange, *scanone*–green, *fitqtl*: additive–black, *fitqtl*: epistatic–gray, EM-algorithm–blue (see all LOD score profiles in S6 Fig and the exact coordinates of the markers, recombination events and QTL intervals in S3 Table). (B, D): Phenotype panels for the corresponding QTL analysis are on the left. The probability of being sensitive (white) or insensitive (black) is shown for each individual. Yellow boxes indicate the individuals that were excluded in the “reduced” dataset due to the probability phenotype between 0.3 and 0.7 (see S1 Methods QTL mapping section and S3 Fig). Numbers on the right indicate for each EM panel how many out of 1000 runs that panel was found, and the fraction of individuals in each panel which had an error > 0.10 from the original data (see S1 Methods QTL mapping/EM-pipeline, S3 Table. The green marks the panel with the highest convergence and lowest error.
(PDF)

S9 Fig Testing EM-scanone pipeline by using sex as a known binary phenotype. We set out to test how well the EM-scanone pipeline identifies binary phenotypic panels from starting probabilities with varying degrees of certainty. We used sex as a known binary phenotype and replaced “0” and “1” to simulate different probability scenarios. A: The insensitivity probability phenotypes in 4 mapping families have varying degrees of certainty. To illustrate their distribution we used the local regression fitting “loess method” of R package ggplot2. B-C: We used sex phenotype and genotype matrix of the RxP_F2.1 mapping family, simulated different probability scenarios and ran EM-scanone pipeline. B left: In models A, B and C, a portion of binary phenotypes was kept fixed: 75%, 50%, and 25% respectively. The remaining 25–75% individuals were given a probability phenotype drawn from the linear distribution: individuals with phenotypes 1 were given a score between 0.99 and 0.5, and those with phenotype 0, values between 0.01 and 0.5. In models D-G there were no certain phenotypes. Individuals with phenotype 1 were given scores between 0.99–0.5 (D), 0.87–0.5 (E), 0.75–0.5 (F); while all individuals that originally had a phenotype 0 gained a score between 0.5–0.01 (D), 0.5–0.125 (E), 0.5–0.25 (F), 0.5–0.375 (G). B right: To model a more realistic distribution, we tested the logistic function y = 1/(1+exp(k*(x-0.5))) using k values of 0.2, 0.3, 0.5, 1, 2 and 5. C: Plotted are the QTL intervals and peak positions of the phenotypic panels found in more than 5% of the 1000 runs. The percentage of convergence and the difference from the true sex phenotype are depicted on the right. In model E 58.8% of the runs failed because the pipeline could not identify a panel that gives a higher scanone LOD score from the starting one. This also indicates that this scenario has too much uncertainty for the EM-pipeline to produce credible results and it is a good additional sanity check. The scenarios closest to our insensitivity phenotypes (panel A) are the linear models B-D and logistic models LOG0.3 and LOG0.5.
(PDF)

S10 Fig Sex locus in Ros-2FMxPor-1SL and Jean-2NMxPor-1SL. To complement the S9 Fig. and validate the resolved Jean-2NMxPor-1SL genotype matrix (see S1 Methods: QTL mapping: Informative variants and genotype matrix in Jean-2NM x Por-1SL), we identified the sex locus in the four mapping families: (A)RPxR-BC.1, (B) (RxP-F2.1, (C) JxP JxP-F2.1.6, (D) JxP-F2.2 .3. The QTL intervals are listed in S10 Table.
(PDF)

S11 Fig BayPass analysis for Ros-2FM dataset. (A) The Bayesian factor (BFis) showing the strength of the association is plotted against the differentiation measure (XtXst) for all polymorphic variants analyzed by BayPass. 357 significantly associated SNPs and indels (BFis > 20, eBPis > 2, XtXst > 21.67) are depicted in red. (B) Kinship matrix Ω is given as a heatmap showing reconstructed relationships between nine tested populations. (C-D) The effect of 357 associated variants was analyzed by SNPeff. (C) The effects of the variants on the surrounding genes are depicted in a pie chart. (D) The estimated impact of 357 associated variants is represented in a pie chart.
(PDF)

S12 Fig SNPs associated with the loss of sensitivity to tidal turbulence in Ros-2FM. QTL mapping and association mapping was performed to determine the most likely causative mutations underlying the phenotypic loss in Ros-2FM. Nine loci were identified (Fig 3). Although STAT1 locus was the most likely causal one (Fig 3C), we investigated all other polymorphisms underlying the QTLs on the second and third chromosomes. A-H panels show for each of these loci: gene affected by the associated SNPs (blue gene models), association score (BFis), genomic differentiation (Fst) between Ros-2FM and Ros-2NM, and selective-sweep analysis (normalized XP-nSL). Phylogenetic trees of all 15 candidate genes (blue gene models) can be seen in S13 and S14 Figs.
(PDF)

S13 Fig Phylogenetic trees of all the potential candidate genes on the second chromosome. Species are color-coded and represented by a pictogram next to the gene names: outgroup–gray, *Caenorhabditis elegans*–green, *Drosophila melanogaster–*orange, *Anopheles gambiae*–brown, *Mus musculus*–light blue, *Homo sapiens*–dark blue, *Clunio marinus* candidate gene–red, *Clunio marinus* other orthologs of the candidate gene–black. Bootstrap values are written above each node. The estimated distance is given below each tree. (A) Protein kinase regulatory subunits. (B) Heparan sulfate proteoglycan Perlecan / Terribly reduced optic lobe. (C) Alan shepard. (D) Signal transducer and transcription activator. (E) Unnamed gravitaxis gene. (F) Lon protease mitochondrial.
(PDF)

S14 Fig Phylogenetic trees of all the potential candidate genes on the third chromosome. Species are color-coded and represented by a pictogram next to the gene names: outgroup–gray, *Caenorhabditis elegans*–green, *Drosophila melanogaster–*orange, *Anopheles gambiae*–brown, *Mus musculus*–light blue, *Homo sapiens*–dark blue, *Clunio marinus* candidate gene–red, *Clunio marinus* other orthologs of the candidate gene–black. Bootstrap values are written above each node. The estimated distance is given below each tree. (A) Snail-like family of transcription factors. (B) O-acyltransferase family. (C) ATP-dependent RNA helicase me31b. (D) Membralin. (E) Globin family. (F) tRNA splicing endonuclease subunit 34 (Tsen34) (G) Unknown protein. (H) Chico, insulin receptor substrate.
(PDF)

S15 Fig GO term enrichment of genes associated with the loss of sensitivity to tidal turbulence in Ros-2FM. BayPass and SNPeff were used to identify 178 genes associated with the loss of sensitivity to tidal turbulence in Ros-2FM. (A) 51 genes that are driving 78 significant GO terms are depicted. Hierarchical clustering of genes and GO terms reveals major clusters of GO terms (color-coded) and listed in panel (B): yellow = Reproduction, orange = Development & Morphogenesis, brown = Protein & organelle localization, pink = Nervous system, purple = Sensory system, light green = Signaling, dark blue = Circadian, light blue = Metabolic process, dark green = Response, gray = Behavior. (C) Several GO terms related to sensory nervous system and potentially involved in mechanosensory entrainment are given in the table together with the corresponding genes. (D) Venn diagram is showing the number of genes that went into the GO term enrichment analysis.
(PDF)

S16 Fig Calculating probability phenotypes for JxP-F2.1.6 and JxP-F2.2.3. (A-F) The fraction of emerged adults and the mean (red) and median (blue) vectors are plotted. (A) Por-1SL strain. (B) Jean-2NM strain. (C-D) F1 progenies of the two mapping families (E-F) F2 progenies of the two mapping families. (G-H) Kernel density estimates for parental, F1, and F2 generations for each of the two mapping families. Both F2 progenies show a phase shift as compared to the parental Por-1SL strain. (I-J) Bar graphs depict probabilities of finding sensitive—Por-1SL-like (white) or insensitive—Jean-2NM-like (black) individuals on each day in the two crossing families JxP-F2.1.6 (I) JxP-F2.2 .3 (J).
(PDF)

S17 Fig Jean-2NMxPor-1SL QTL mapping analysis. Complete QTL mapping results for the two crossing families: JxP-F2.1.6 and JxP-F2.2.3. (A, H) Bar graphs show the number of emerged individuals per day. The predicted ratio of insensitive (black) and sensitive (white) individuals is plotted. (B, I) LOD scores of interval mapping analysis (*scanone*) are designed to detect additive QTLs. The significance threshold (dashed line) was estimated in 1000 permutations with a 5% cutoff. (C, J) CIM analysis with backward regression method, 5 control markers, and a window size of 10 cM. Threshold values are given in S8 Table. (D, K): LOD scores of *scanone* on EM-optimized binary phenotypes. Results are shown for panels obtained in at least 5% of the cases in 1000 runs (S8 Table). The significance threshold (dashed line) was estimated in 1000 permutations with a 5% cutoff. (E, L) LOD scores of significant QTLs in multiple QTL mapping pipeline (*fitqtl*). Black lines: additive QTLs, gray lines: QTLs in epistasis. p-value of F statistic is marked: * p-value < 0.05; ** p-value of <0.01. *Fitqlt* statistics are given in S8 Table. (F, M) Confidence intervals and for the full QTL analysis. QTL intervals: composite interval mapping–orange, *scanone*–green, *fitqtl*: additive–black, *fitqtl*: epistatic–gray, EM-algorithm–blue (exact coordinates of the markers in S8 Table. (G, N): Phenotype panels for the corresponding QTL analysis. The probability of being sensitive (white) or insensitive (black) is shown for each individual. Numbers on the right indicate for each EM panel how many out of 1000 runs that panel was found, and the fraction of individuals in each panel which had an error > 0.10 from the original data (see methods QTL mapping/EM-pipeline, S8 Table). The green marks the panel with the highest convergence and lowest error.
(PDF)

S18 Fig Probability of finding sensitive and insensitive individuals in several Jean-2NMxPor-1SL intercross families. (A-E left and middle) The fraction of emerged adults per generation is shown on a circular plot together with the mean and median vector. (A). Jean-2NM parental strain (B) Por-1SL parental strain. (B-E left) Independent F1 progenies of the four intercross families JxP-F2.1–4. (B-E middle) Combined emergence of several F2 families of four intercross families JxP-F2.1–4. Number of individuals is given in S2 Table. (B-E right) Bar graphs show probabilities of finding sensitive Por-1SL-like (white) or insensitive Jean-2NM-like (black) individuals on each day in the four intercross families.
(PDF)

S19 Fig BayPass analysis for Jean-2NM dataset. Association analysis was performed to find mutations associated with the loss of sensitivity to tidal turbulence in the Jean-2NM population. (A) Median vector length was used as a proxy for sensitivity to this cue (S1 and S5 Tables), (solid lines with arrows; values outside the circle). (B) Association analysis for median vector length with 769.379 SNPs and small indels. Bayesian factor (BFis) is plotted for each variant along the three chromosomes. (C) We found 173 significantly associated SNPs and indels (BFis > 20, eBPis > 2, XtXst > 20.02; see Methods section for details) marked in red. A list of effects and genes affected by mutations is given in S9 Table. (D) Kinship matrix Ω is given as a heatmap showing reconstructed relationships between nine tested populations. (E-F) The effect of 173 associated variants was analyzed by SNPeff. (E) The effects of the variants on the surrounding genes are depicted in a pie chart. (F) The estimated impact of 173 associated variants is represented in a pie chart.
(PDF)

S1 Table The origin of Clunio populations and their entrainment to tidal turbulence. (XLSX)

S2 Table Crossing families. (XLSX)

S3 Table Ros-2FMxPor-1SL QTL mapping. (XLSX)

S4 Table Whole genome sequencing details and sampling sites. (XLSX)

S5 Table BayPass phenotype. (XLSX)

S6 Table BayPass results—Insensitivity to tidal turbulence in Ros-2FM. (XLSX)

S7 Table Insensitivity to tidal turbulence in Ros-2FM–Gravitaxis. (XLSX)

S8 Table Jean-2NMxPor-1SL QTL mapping. (XLSX)

S9 Table BayPass results—Insensitivity to tidal turbulence in Jean-2NM. (XLSX)

S10 Table RAD sequencing primers and adapters. (XLSX)

S11 Table Informative variants for QTL mapping. (XLSX)

S12 Table QTL mapping—Sex_Locus. (XLSX)

S1 Script Phenotyping. (ZIP)

S2 Script Qtl Input Pipeline. (ZIP)

S3 Script Qtl EM Scanone. (ZIP)

S4 Script Vcf Update Cluma. (ZIP)

S1 Methods Supplemental methods. (DOCX)
