# Supplementary figures and images for: Genetic analysis of a phenotypic loss in the mechanosensory entrainment of a circalunar clock
Source: PLoS Genet. 2023 Jun 22;19(6):e1010763. doi: 10.1371/journal.pgen.1010763 (PMC10286985; doi:10.1371/journal.pgen.1010763)

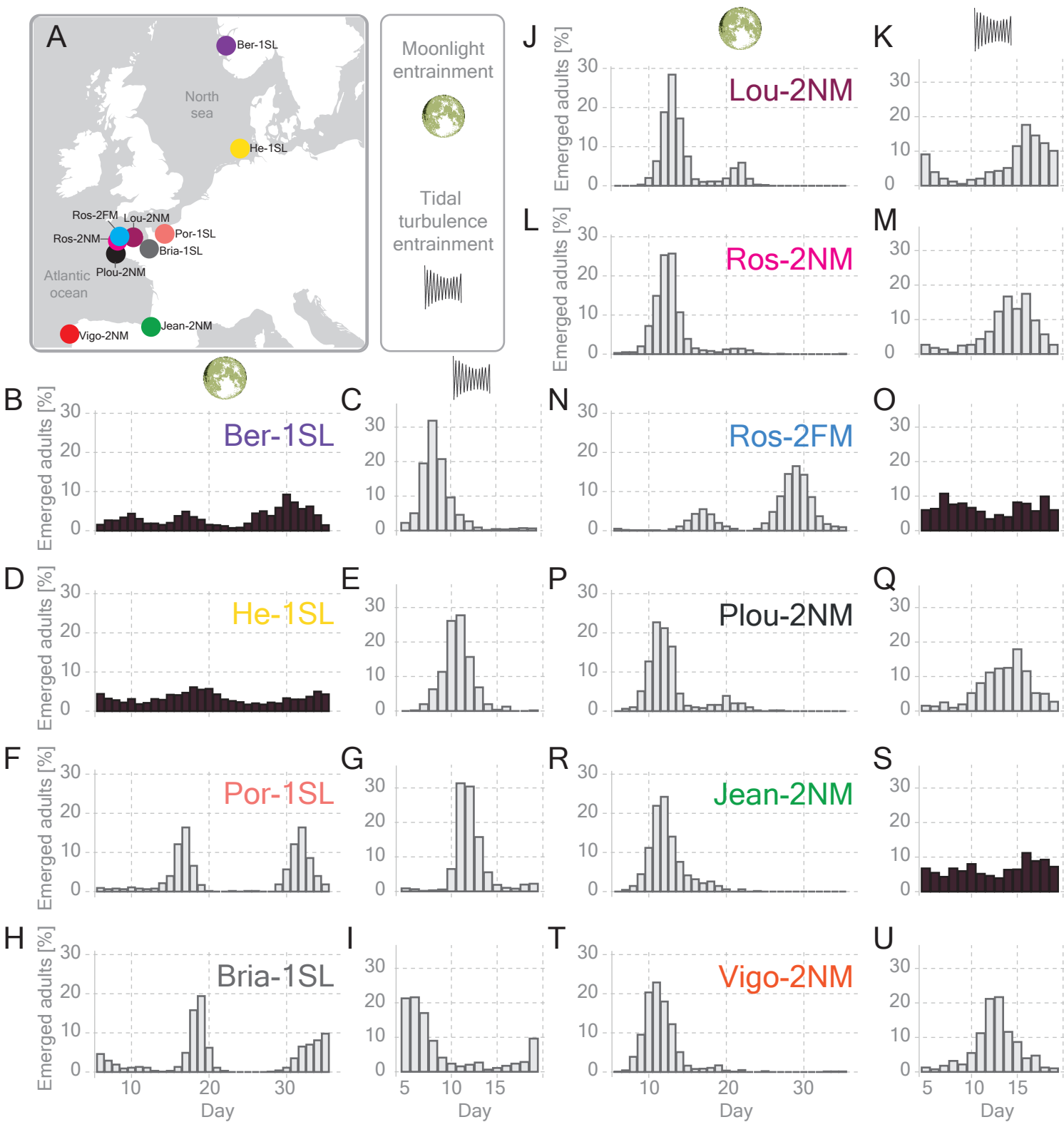

S.Figure 1

Supplement: S1 Fig — Clunio populations are differentially sensitive to moonlight or tidal turbulence (A) Origin of the Clunio strains. Strains are color-coded and their names are depicted in the body of the graph. (B-U) Graphs show the fraction of emerged individuals entrained under laboratory conditions by either artificial moonlight (four nights of light every 30 days) or tidal turbulence (vibration of ~50 Hz 30dB above background noise in 6h 10min ON– 6h 15min OFF intervals resulting in a 15-day pattern). The total number of individuals, exact names of geographical locations, and the year when strains were established are given in S1 Table. Strains differ in the period, phase of emergence, and sensitivity to the synchronizers. The emergence of strains considered insensitive to moonlight or tidal turbulence is marked in black. Map data was obtained from https://www.naturalearthdata.com/downloads/50m-physical-vectors/. (PDF) [file pgen.1010763.s001.pdf]

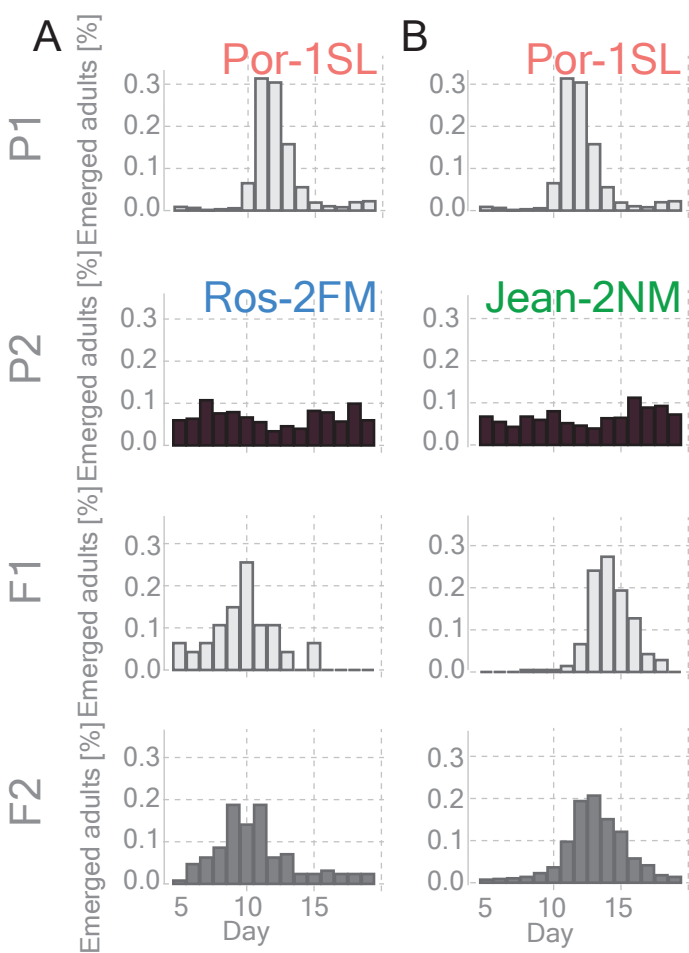

**S.Figure 2**

Supplement: S2 Fig — Crossing experiments were performed to assess the inheritance of sensitivity to tidal turbulence. Graphs show fractions of emerged adults per day in parental populations, F1 and F2 (F1xF1) generations. (A) Intercross between Por-1SL and Ros-2FM. (B) Intercross between Por-1SL and Jean-2NM. The total number of individuals per generation is listed in S2 Table. The color of the bars represents increasing levels of sensitivity to tidal turbulence from sensitive (light gray) to insensitive (black). (PDF) [file pgen.1010763.s002.pdf]

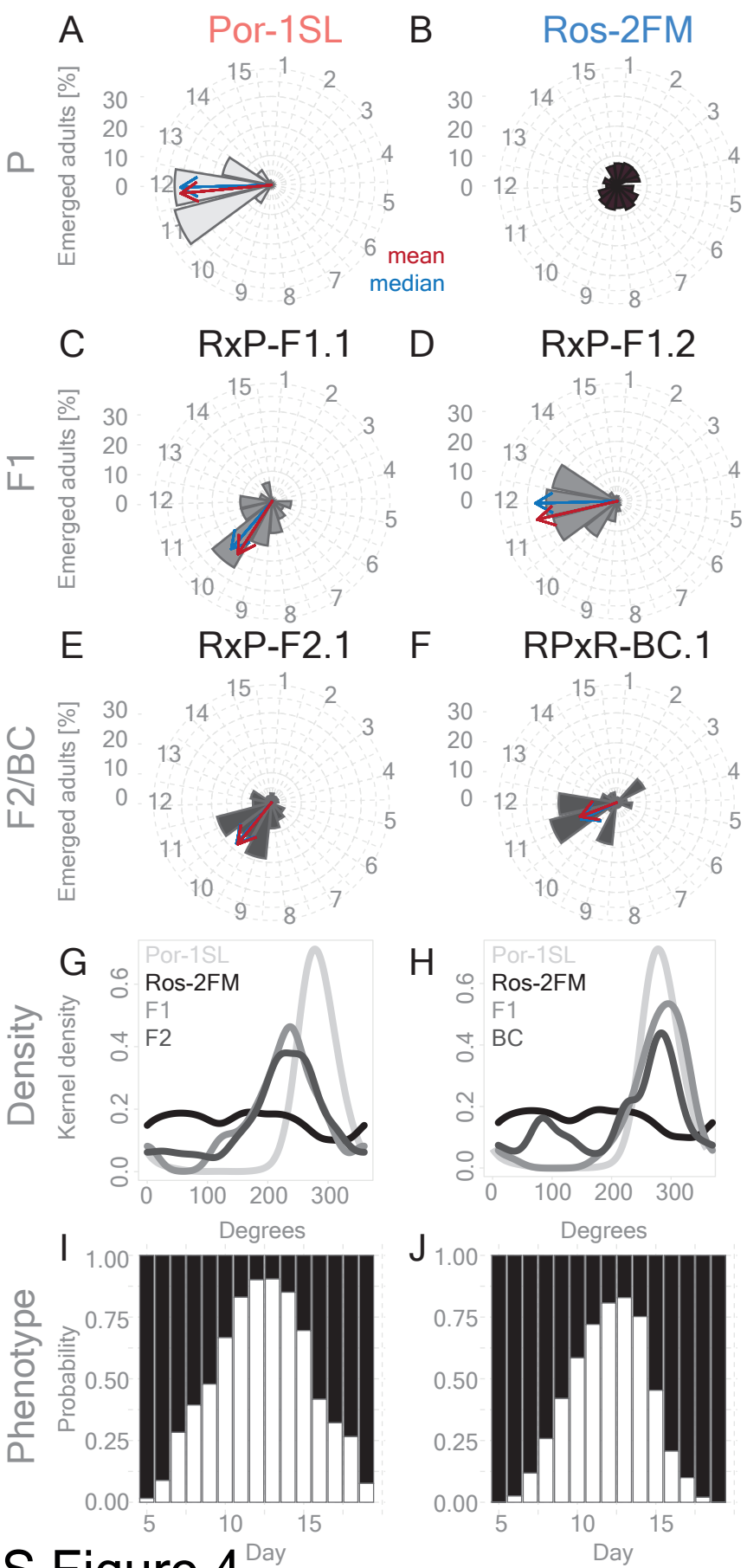

Supplement: S4 Fig — (A-F) The fraction of emerged adults per generation is shown on a circular plot together with the mean and median vectors. (A) Por-1SL strain. (B) Ros-2FM strain. (C) RxP-F1.1 and RxP-F2.1 generation. (D) RxP-F1.2 three crossing families were raised together (gave rise to RPxR-BC.1). (E) RxP-F2.1 is a F1-24 x F1-24 intercross. (F) RPxR-BC.1 is a backcross of an F1.2 individual to Ros-2FM. (G-H) Kernel density estimates for parental, F1, and F2/BC generations for each of the two mapping families. RxP-F2.1 crossing family shows a 2-day phase shift as compared to Por-1SL. RPxR-BC.1 crossing family does not show considerable phase-shift. (I-J) Bar graphs show probabilities of finding sensitive Por-1SL-like (white) or insensitive Ros-2FM-like (black) individuals on each day in the two crossing families RxP-F2.1 (I) and RPxR-BC.1 (J). (PDF) [file pgen.1010763.s004.pdf]

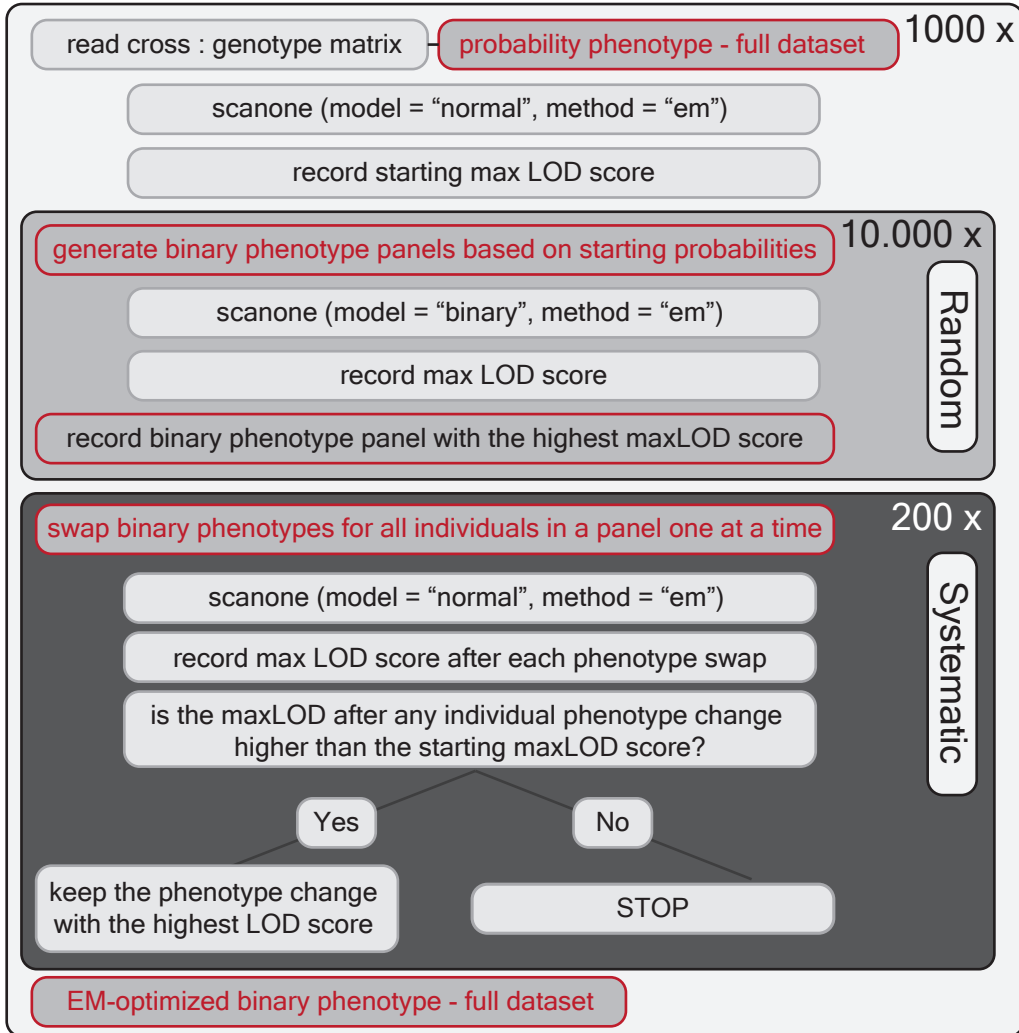

S.Figure 5

Supplement: S5 Fig — An EM algorithm was designed to generate optimized binary phenotype panels to a crossing family given the calculated probability of finding insensitive individuals on each experimental day. For a detailed explanation see the S1 Methods/QTL mapping/EM Expectation-maximization (EM) algorithm paragraph. (PDF) [file pgen.1010763.s005.pdf]

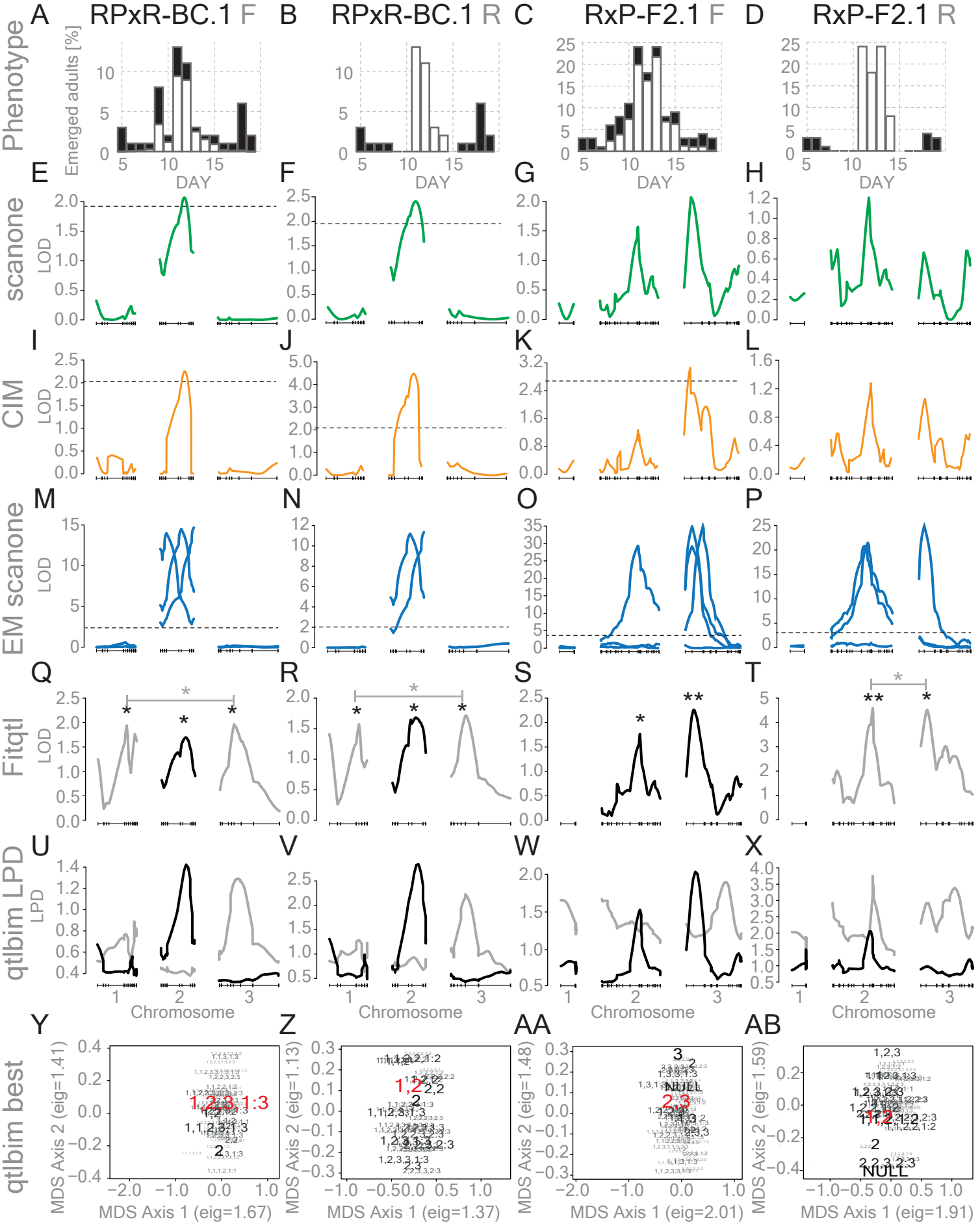

S.Figure 6

Supplement: S6 Fig — Complete QTL mapping results for the two crossing families (RxP-F2.1 and RPxR-BC.1) and two datasets each (F = full and R = reduced) are given. (A-D) Bar graphs show the number of emerged individuals per day. The predicted ratio of insensitive (black) and sensitive (white) individuals is plotted. (E-H) LOD scores of interval mapping analysis (scanone) are designed to detect additive QTLs. Probability phenotypes were used for full datasets and binary phenotypes for reduced datasets. The significance threshold (dashed line) was estimated in 1000 permutations with a 5% cutoff. (I-L) CIM analysis with backward regression method, 5 control markers, and a window size of 10 cM. Threshold values are given in S3 Table. (M-P): LOD scores of scanone on EM-optimized binary phenotypes. Results are shown for panels obtained in at least 5% of the cases in 1000 runs (S3 Table). The significance threshold (dashed line) was estimated in 1000 permutations with a 5% cutoff. (Q-T) LOD scores of significant QTLs in multiple QTL mapping pipeline (fitqtl). Black lines: additive QTLs, gray lines: QTLs in epistasis. p-value of F statistic is marked: * p-value < 0.05; ** p-value of <0.01. Fitqlt statistics are given in S3 Table. (U-AB) To find the best model for multiple QTL mapping (fitqtl) we also tried the qtlbim package. (U-X) We first ran LPD (Log Posterior Density) scan that uses Bayesian model averaging to explore the most probable models. Most likely additive QTLs are shown in black and most likely QTLs in epistasis are shown in gray. (Y-AB) The “best” function of the qtlbim package then selects the most probable model. The larger the font size the larger posterior probability the pattern has. The 2-D multidimensional scaling (MDS) projection is based on the square of the attenuation. If the loci agree exactly, there is no attenuation. The best model is marked in red. The numbers represent chromosomes. (U) The best model for the RPxR-BC.1 full dataset contains 3 QTLs and one epistatic [file pgen.1010763.s006.pdf]

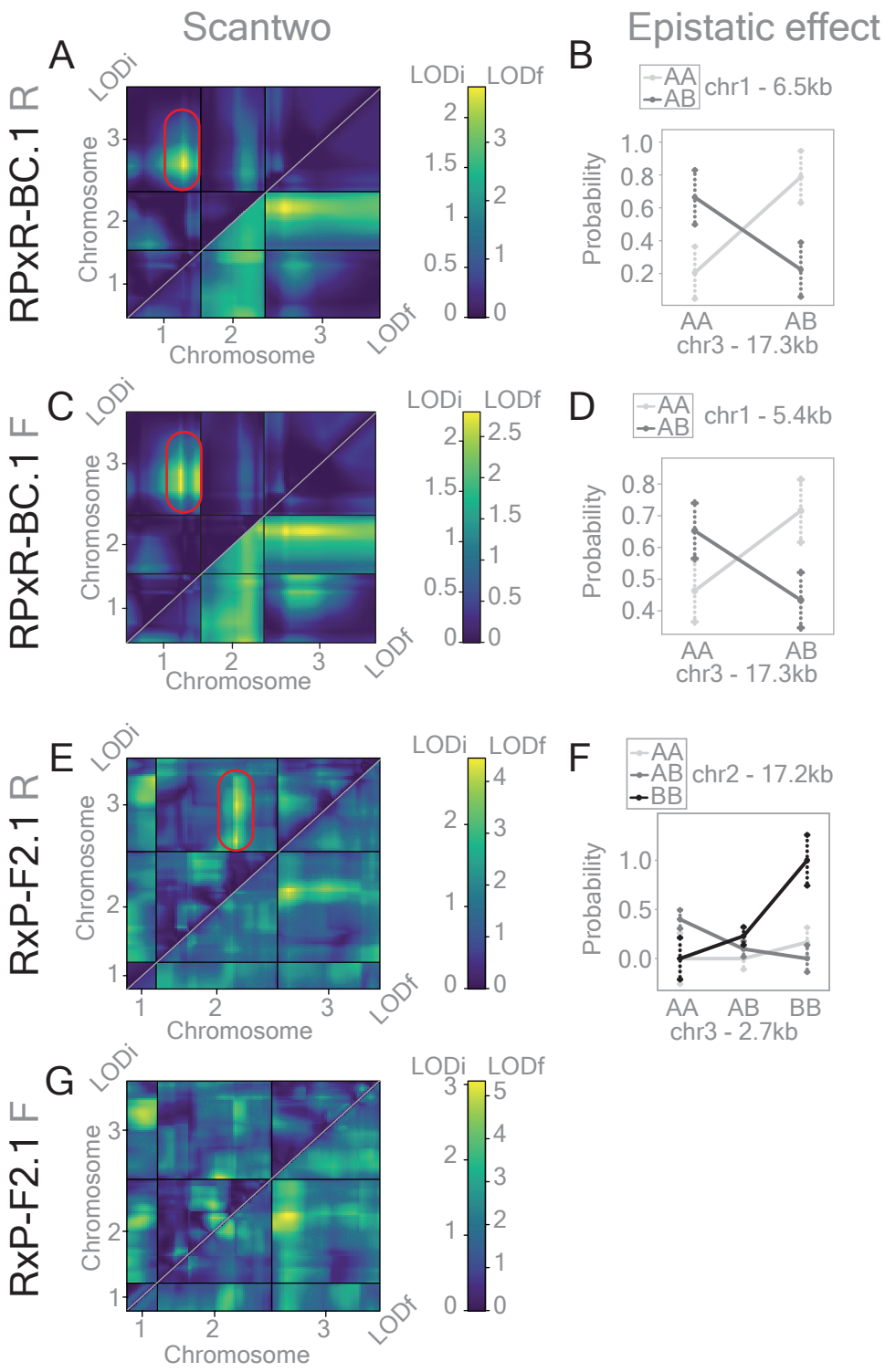

S.Figure 7

Supplement: S7 Fig — To scan for QTLs in epistasis, we used the scantwo function (rqtl package). Datasets are labeled (left gray; F = Full and R = Reduced) and correspond to S6 and S8 Figs. (A, C, E, G): Scantwo heatmaps for three chromosomes show LODf in the lower right corner that measures the improvement in the fit of the full two-locus model over the null model and indicates the evidence for at least one QTL with allowance for interaction. LODi heatmap is plotted in the upper left corner and measures the improvement in the fit of the full model over that of the additive model, and so indicates evidence for an interaction. Significant QTL epistatic interaction is marked by a red circle. (B, D, F): The epistatic effect for each of the significant interactions on the left is shown. The marker that shows the strongest interaction on each chromosome was selected and its location is marked in gray letters. (PDF) [file pgen.1010763.s007.pdf]

## A RPxR-BC.1

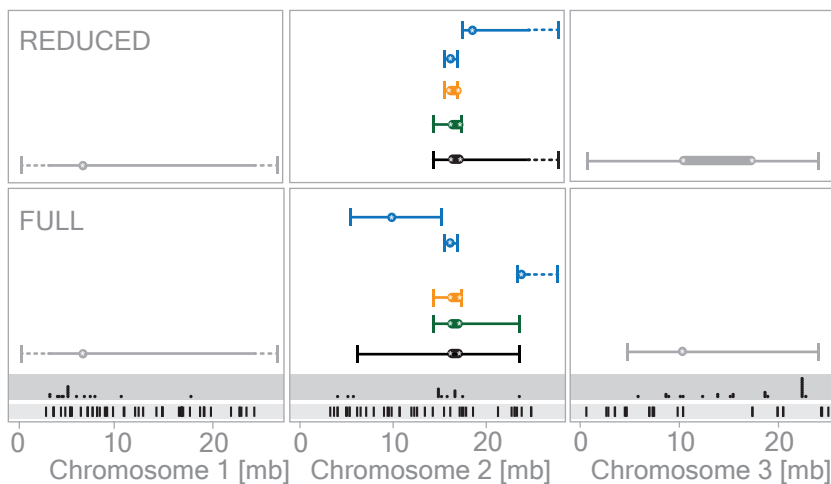

## B

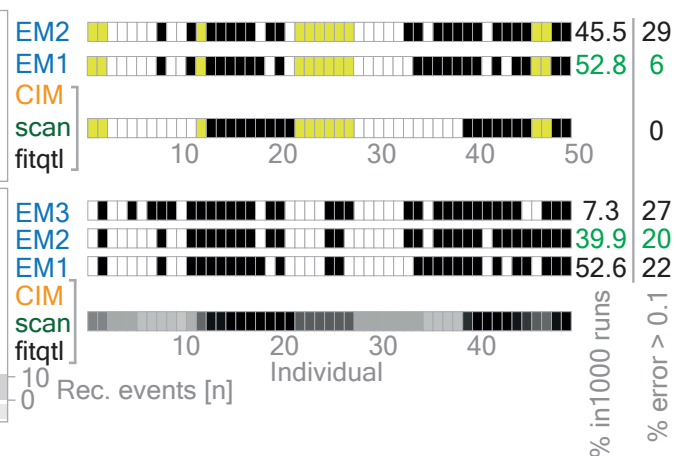

## C RxP-F2.1

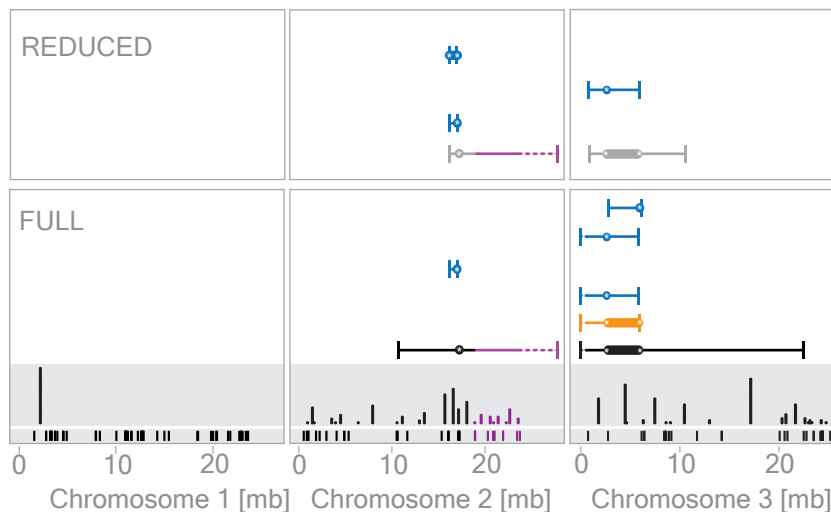

## D

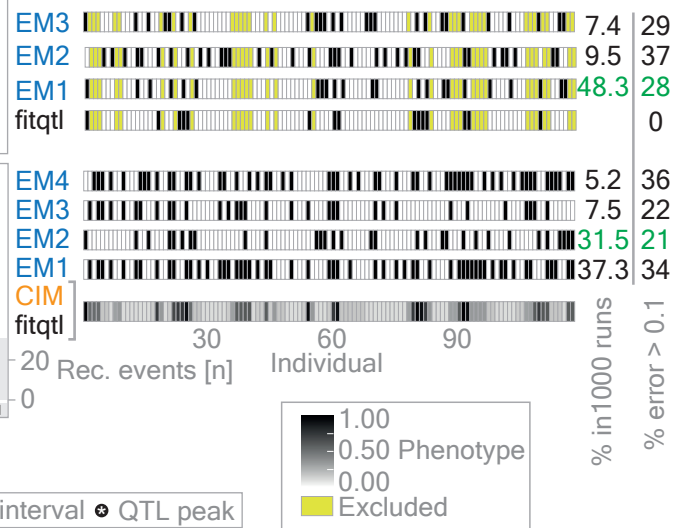

S.Figure 8

Supplement: S8 Fig — Plotted are QTL intervals and phenotype panels for the QTL analysis (S6 Fig). (A, C) QTL intervals: composite interval mapping–orange, scanone–green, fitqtl: additive–black, fitqtl: epistatic–gray, EM-algorithm–blue (see all LOD score profiles in S6 Fig and the exact coordinates of the markers, recombination events and QTL intervals in S3 Table). (B, D): Phenotype panels for the corresponding QTL analysis are on the left. The probability of being sensitive (white) or insensitive (black) is shown for each individual. Yellow boxes indicate the individuals that were excluded in the “reduced” dataset due to the probability phenotype between 0.3 and 0.7 (see S1 Methods QTL mapping section and S3 Fig). Numbers on the right indicate for each EM panel how many out of 1000 runs that panel was found, and the fraction of individuals in each panel which had an error > 0.10 from the original data (see S1 Methods QTL mapping/EM-pipeline, S3 Table. The green marks the panel with the highest convergence and lowest error. (PDF) [file pgen.1010763.s008.pdf]

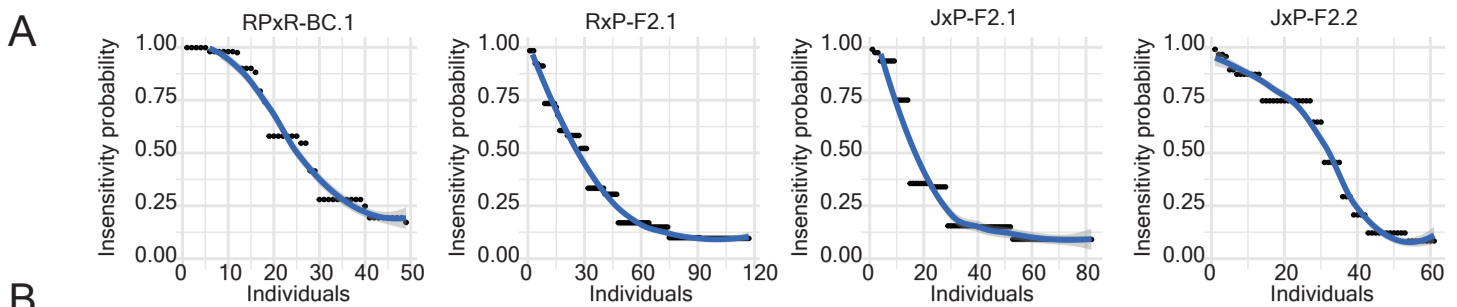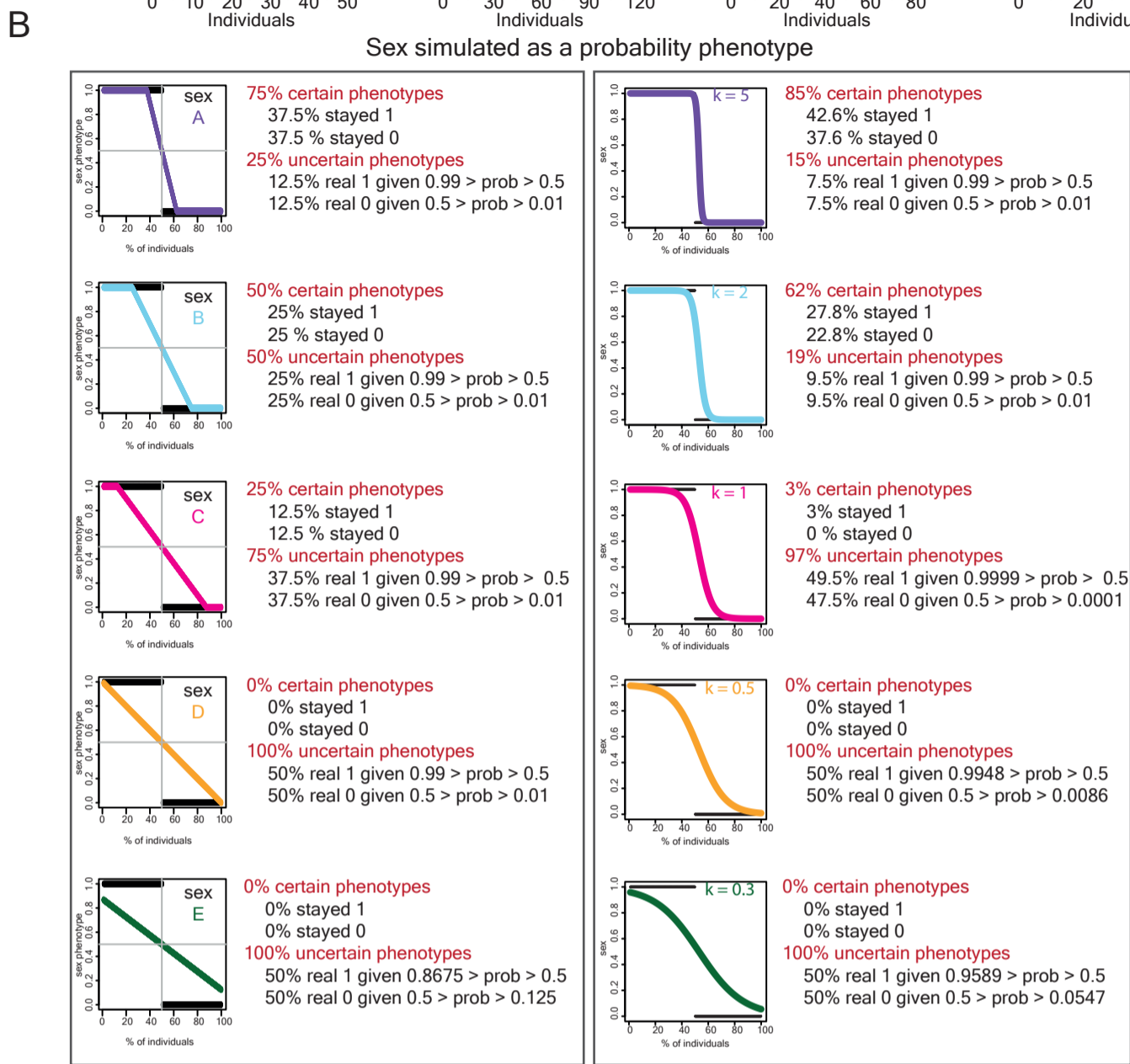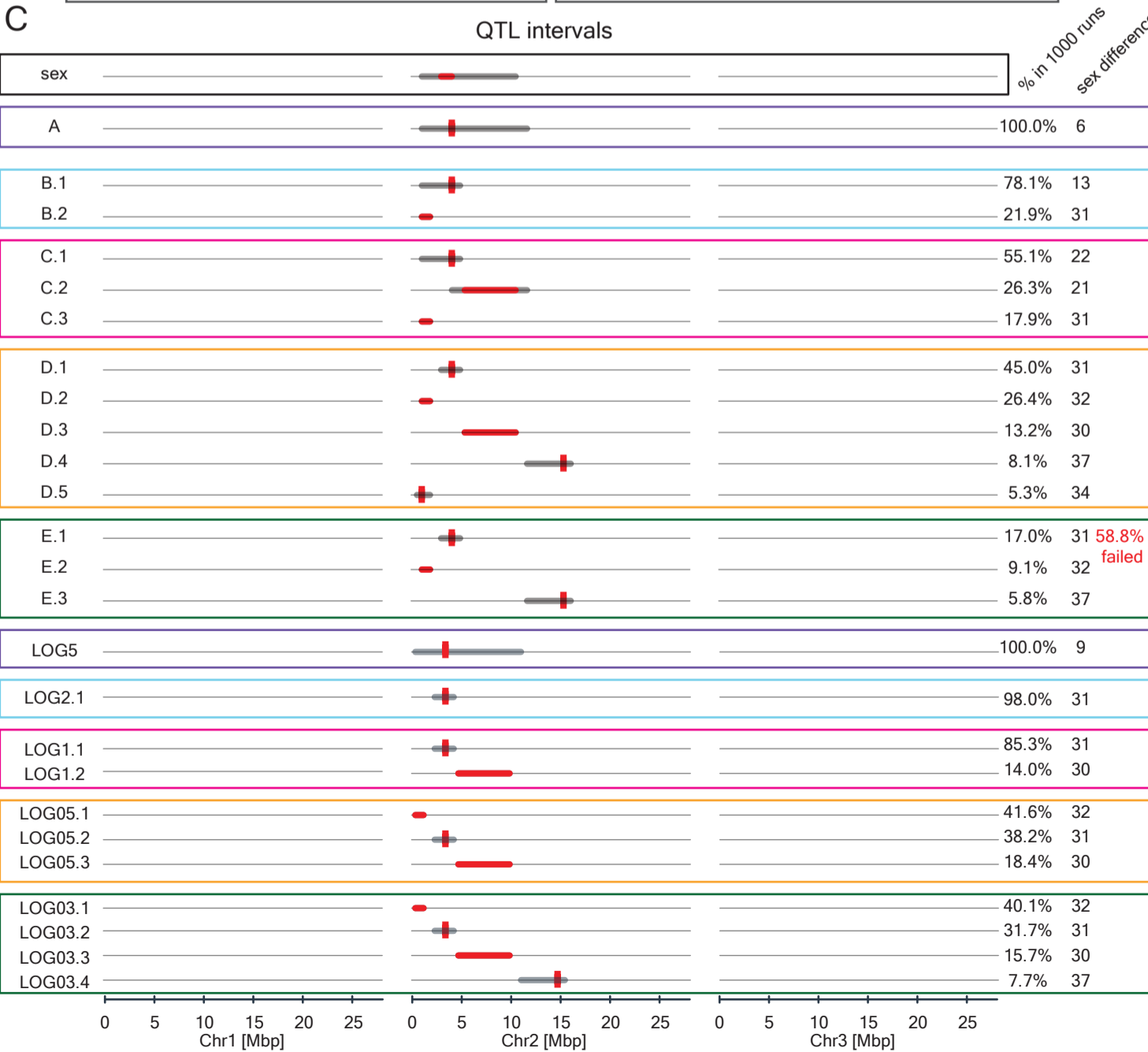

S.Figure 9

Supplement: S9 Fig — We set out to test how well the EM-scanone pipeline identifies binary phenotypic panels from starting probabilities with varying degrees of certainty. We used sex as a known binary phenotype and replaced “0” and “1” to simulate different probability scenarios. A: The insensitivity probability phenotypes in 4 mapping families have varying degrees of certainty. To illustrate their distribution we used the local regression fitting “loess method” of R package ggplot2. B-C: We used sex phenotype and genotype matrix of the RxP_F2.1 mapping family, simulated different probability scenarios and ran EM-scanone pipeline. B left: In models A, B and C, a portion of binary phenotypes was kept fixed: 75%, 50%, and 25% respectively. The remaining 25–75% individuals were given a probability phenotype drawn from the linear distribution: individuals with phenotypes 1 were given a score between 0.99 and 0.5, and those with phenotype 0, values between 0.01 and 0.5. In models D-G there were no certain phenotypes. Individuals with phenotype 1 were given scores between 0.99–0.5 (D), 0.87–0.5 (E), 0.75–0.5 (F); while all individuals that originally had a phenotype 0 gained a score between 0.5–0.01 (D), 0.5–0.125 (E), 0.5–0.25 (F), 0.5–0.375 (G). B right: To model a more realistic distribution, we tested the logistic function y = 1/(1+exp(k*(x-0.5))) using k values of 0.2, 0.3, 0.5, 1, 2 and 5. C: Plotted are the QTL intervals and peak positions of the phenotypic panels found in more than 5% of the 1000 runs. The percentage of convergence and the difference from the true sex phenotype are depicted on the right. In model E 58.8% of the runs failed because the pipeline could not identify a panel that gives a higher scanone LOD score from the starting one. This also indicates that this scenario has too much uncertainty for the EM-pipeline to produce credible results and it is a good additional sanity check. The scenarios closest to our insensitivity phenotypes (panel A) are the linear models B [file pgen.1010763.s009.pdf]

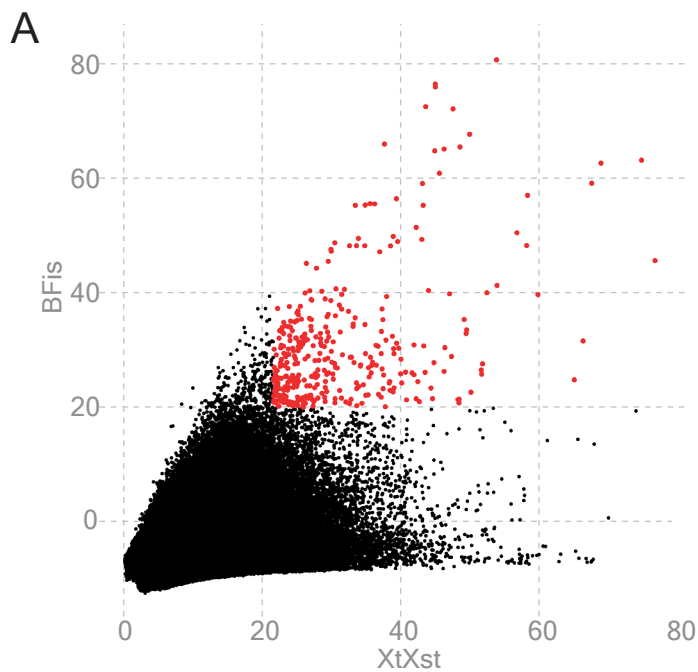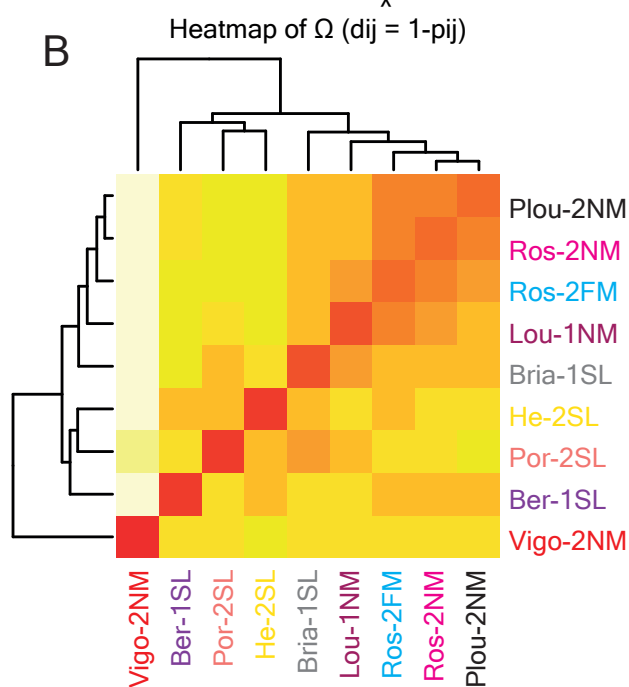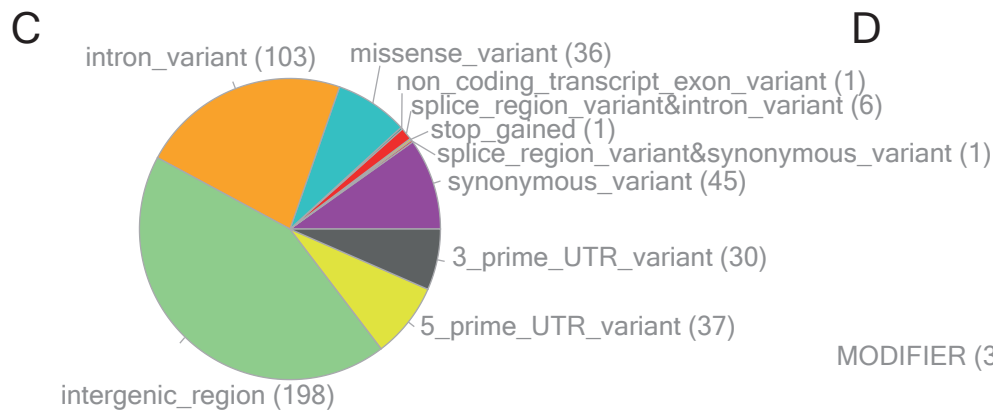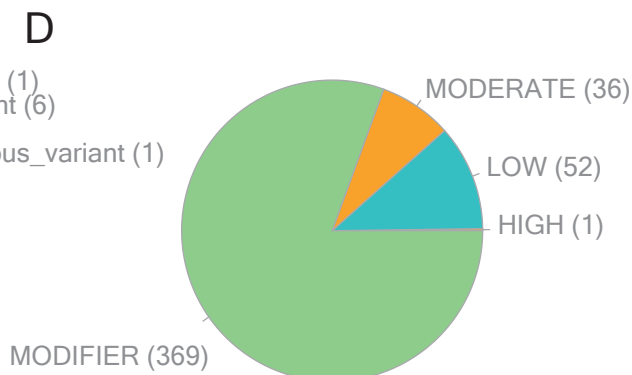

**S.Figure 11**

Supplement: S11 Fig — (A) The Bayesian factor (BFis) showing the strength of the association is plotted against the differentiation measure (XtXst) for all polymorphic variants analyzed by BayPass. 357 significantly associated SNPs and indels (BFis > 20, eBPis > 2, XtXst > 21.67) are depicted in red. (B) Kinship matrix Ω is given as a heatmap showing reconstructed relationships between nine tested populations. (C-D) The effect of 357 associated variants was analyzed by SNPeff. (C) The effects of the variants on the surrounding genes are depicted in a pie chart. (D) The estimated impact of 357 associated variants is represented in a pie chart. (PDF) [file pgen.1010763.s011.pdf]

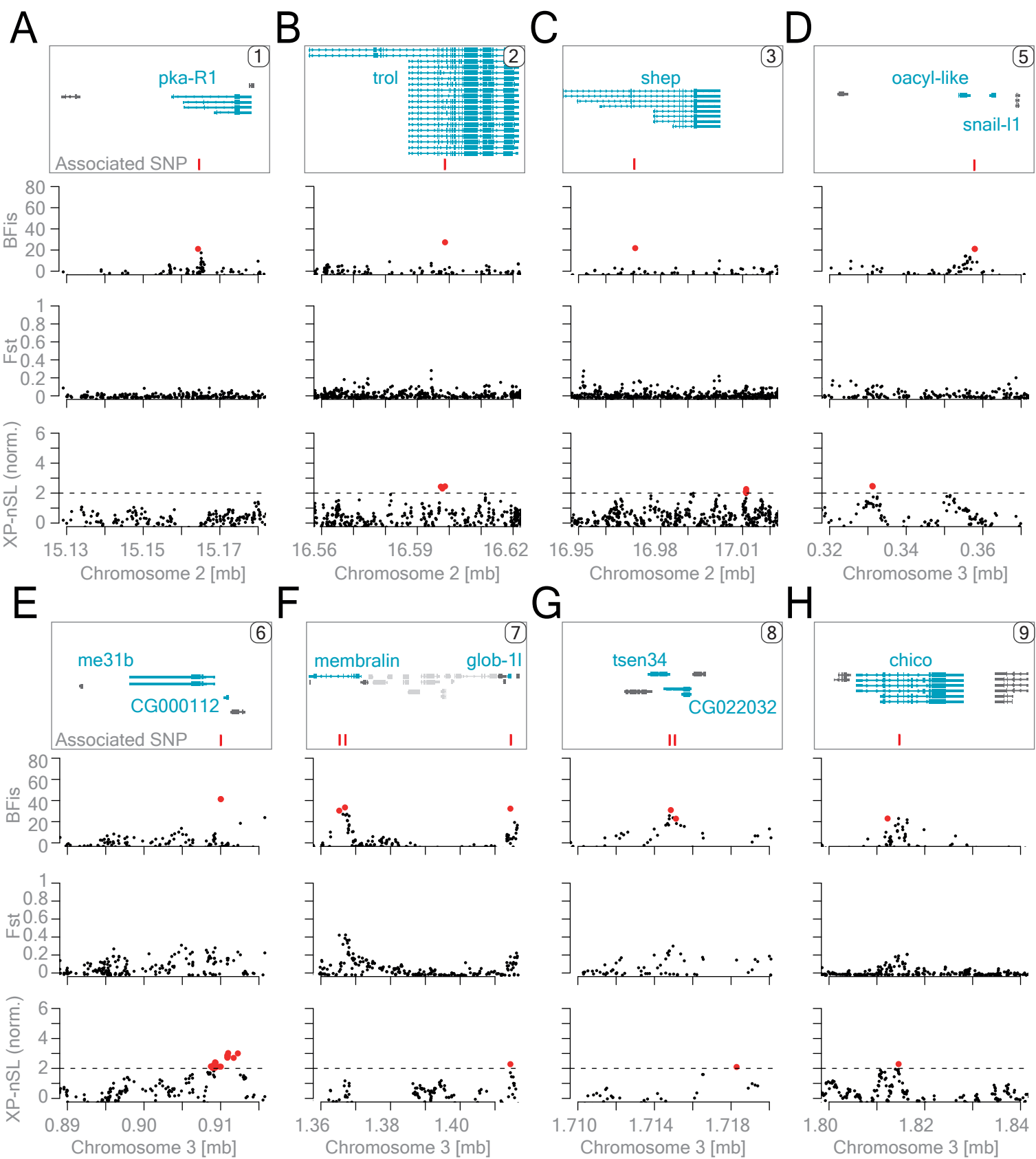

**S. Figure 12**

Supplement: S12 Fig — QTL mapping and association mapping was performed to determine the most likely causative mutations underlying the phenotypic loss in Ros-2FM. Nine loci were identified (Fig 3). Although STAT1 locus was the most likely causal one (Fig 3C), we investigated all other polymorphisms underlying the QTLs on the second and third chromosomes. A-H panels show for each of these loci: gene affected by the associated SNPs (blue gene models), association score (BFis), genomic differentiation (Fst) between Ros-2FM and Ros-2NM, and selective-sweep analysis (normalized XP-nSL). Phylogenetic trees of all 15 candidate genes (blue gene models) can be seen in S13 and S14 Figs. (PDF) [file pgen.1010763.s012.pdf]

A

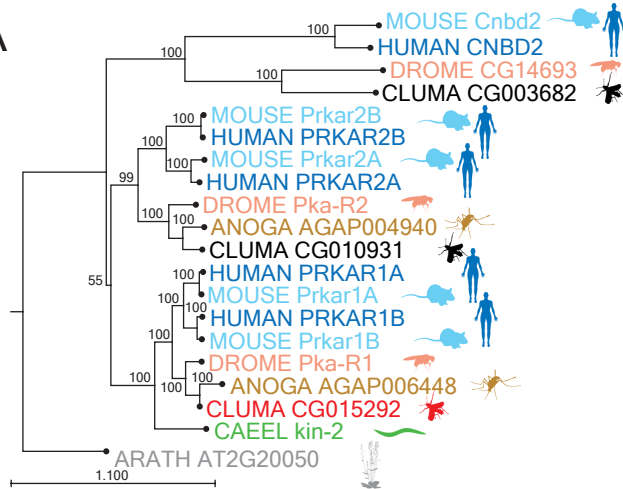

B

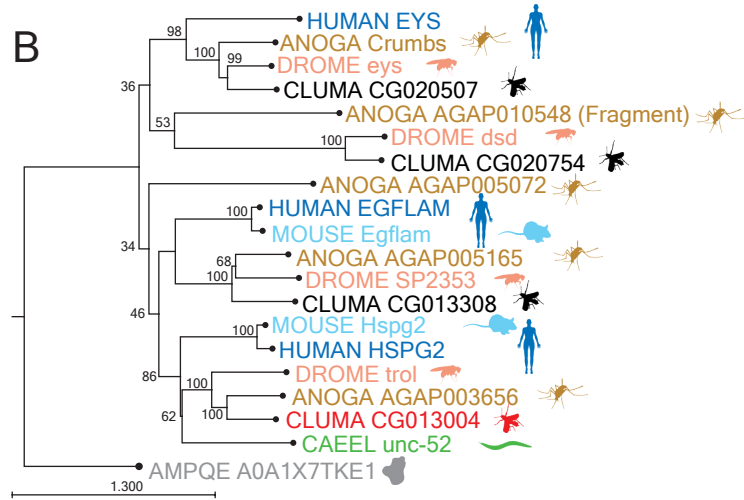

C

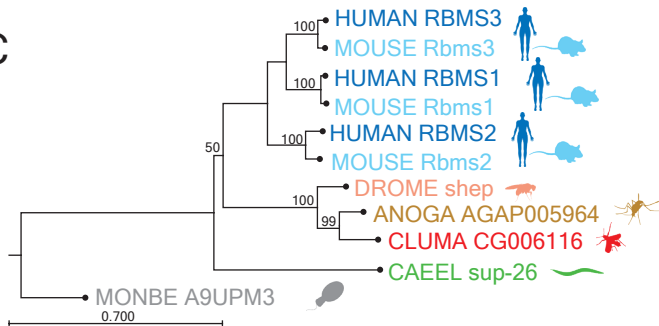

D

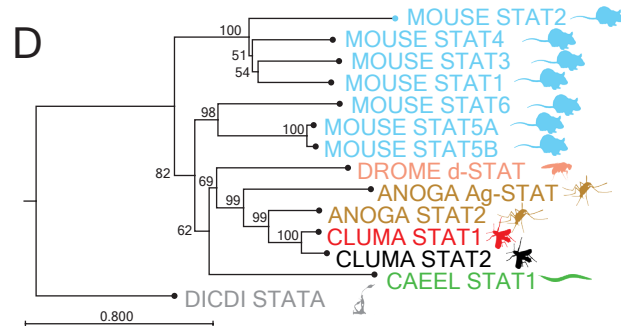

E

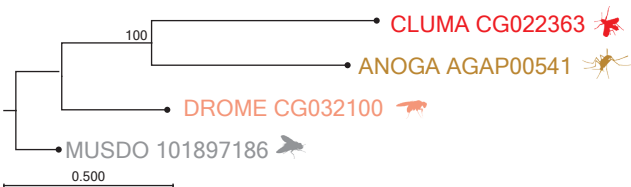

F

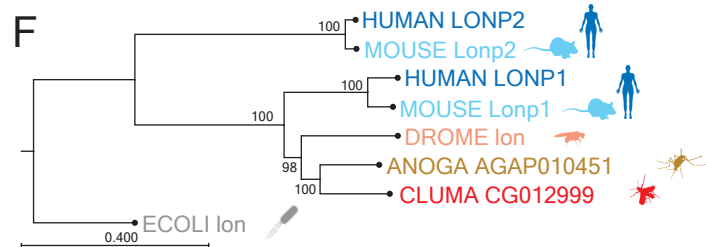

S.Figure 13

Supplement: S13 Fig — Species are color-coded and represented by a pictogram next to the gene names: outgroup–gray, Caenorhabditis elegans–green, Drosophila melanogaster–orange, Anopheles gambiae–brown, Mus musculus–light blue, Homo sapiens–dark blue, Clunio marinus candidate gene–red, Clunio marinus other orthologs of the candidate gene–black. Bootstrap values are written above each node. The estimated distance is given below each tree. (A) Protein kinase regulatory subunits. (B) Heparan sulfate proteoglycan Perlecan / Terribly reduced optic lobe. (C) Alan shepard. (D) Signal transducer and transcription activator. (E) Unnamed gravitaxis gene. (F) Lon protease mitochondrial. (PDF) [file pgen.1010763.s013.pdf]

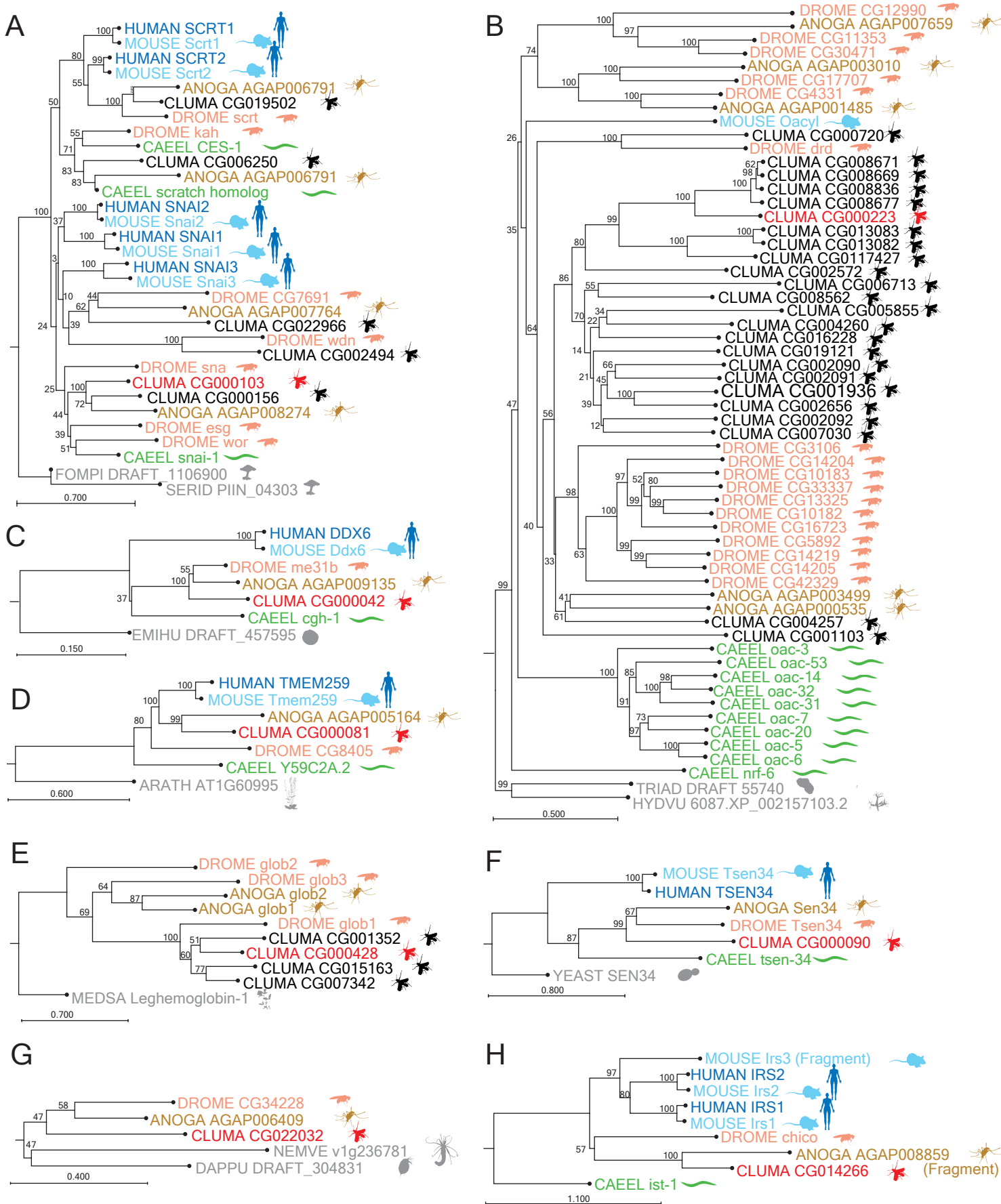

S.Figure 14

Supplement: S14 Fig — Species are color-coded and represented by a pictogram next to the gene names: outgroup–gray, Caenorhabditis elegans–green, Drosophila melanogaster–orange, Anopheles gambiae–brown, Mus musculus–light blue, Homo sapiens–dark blue, Clunio marinus candidate gene–red, Clunio marinus other orthologs of the candidate gene–black. Bootstrap values are written above each node. The estimated distance is given below each tree. (A) Snail-like family of transcription factors. (B) O-acyltransferase family. (C) ATP-dependent RNA helicase me31b. (D) Membralin. (E) Globin family. (F) tRNA splicing endonuclease subunit 34 (Tsen34) (G) Unknown protein. (H) Chico, insulin receptor substrate. (PDF) [file pgen.1010763.s014.pdf]

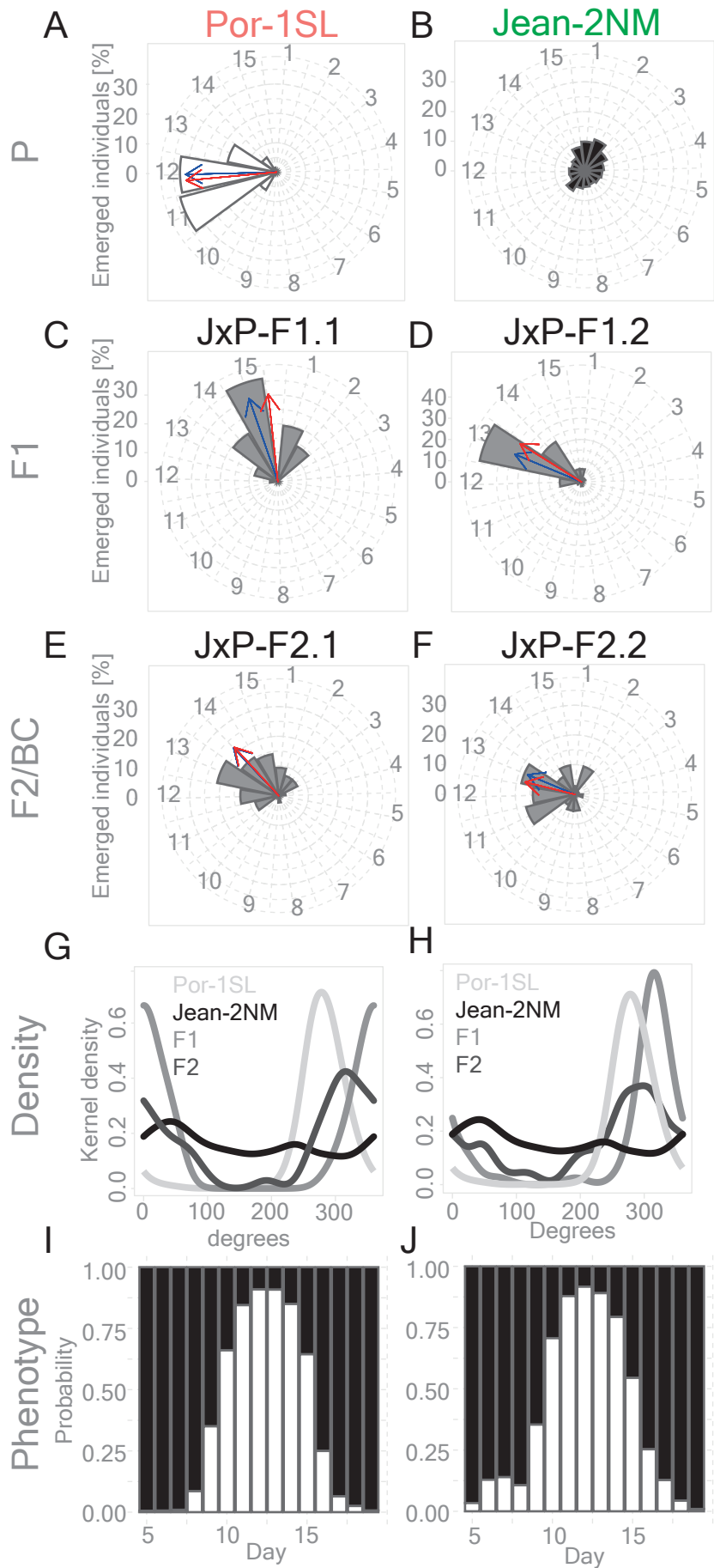

S.Figure 16

Supplement: S16 Fig — (A-F) The fraction of emerged adults and the mean (red) and median (blue) vectors are plotted. (A) Por-1SL strain. (B) Jean-2NM strain. (C-D) F1 progenies of the two mapping families (E-F) F2 progenies of the two mapping families. (G-H) Kernel density estimates for parental, F1, and F2 generations for each of the two mapping families. Both F2 progenies show a phase shift as compared to the parental Por-1SL strain. (I-J) Bar graphs depict probabilities of finding sensitive—Por-1SL-like (white) or insensitive—Jean-2NM-like (black) individuals on each day in the two crossing families JxP-F2.1.6 (I) JxP-F2.2 .3 (J). (PDF) [file pgen.1010763.s016.pdf]

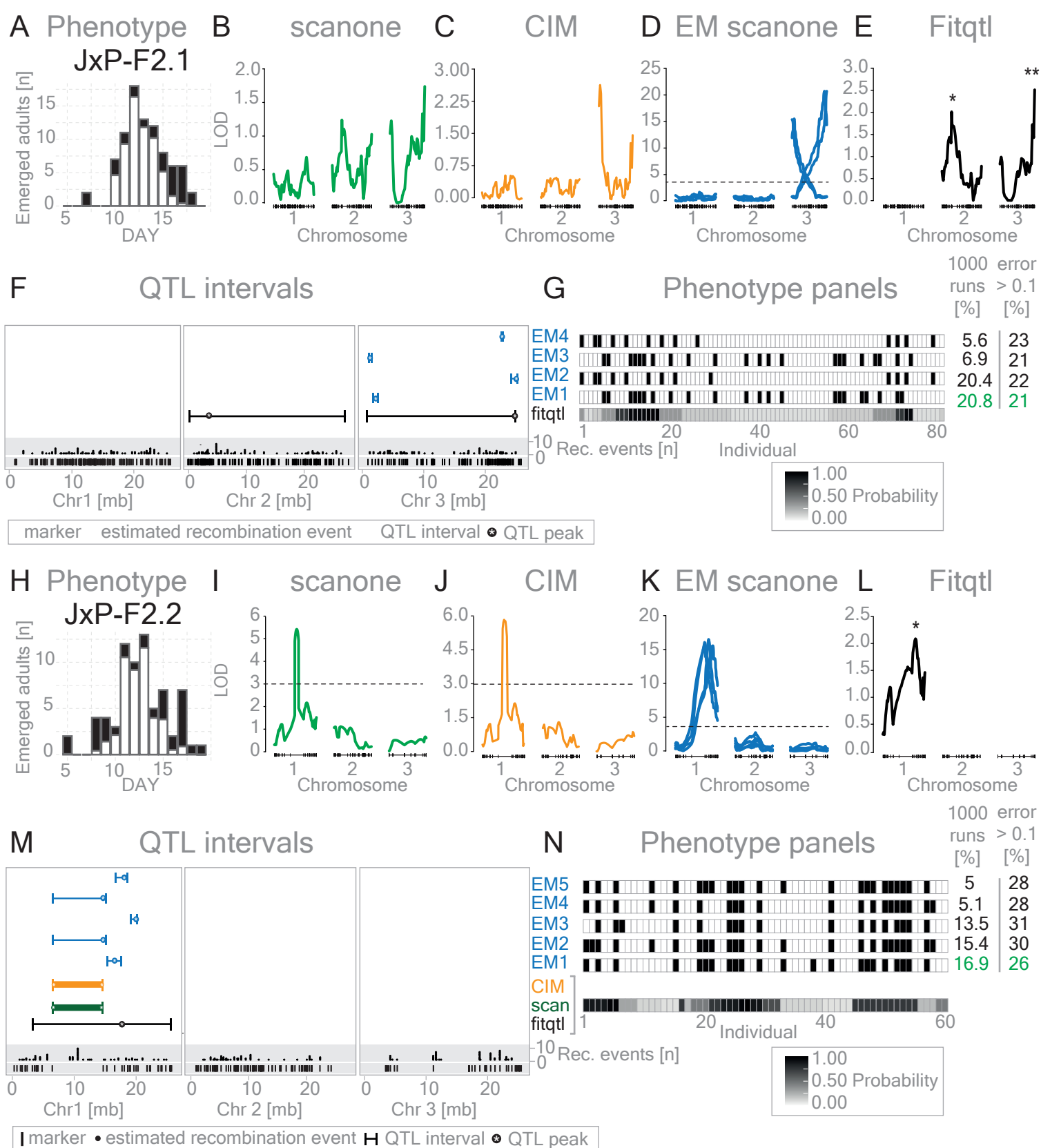

**S.Figure 17**

Supplement: S17 Fig — Complete QTL mapping results for the two crossing families: JxP-F2.1.6 and JxP-F2.2.3. (A, H) Bar graphs show the number of emerged individuals per day. The predicted ratio of insensitive (black) and sensitive (white) individuals is plotted. (B, I) LOD scores of interval mapping analysis (scanone) are designed to detect additive QTLs. The significance threshold (dashed line) was estimated in 1000 permutations with a 5% cutoff. (C, J) CIM analysis with backward regression method, 5 control markers, and a window size of 10 cM. Threshold values are given in S8 Table. (D, K): LOD scores of scanone on EM-optimized binary phenotypes. Results are shown for panels obtained in at least 5% of the cases in 1000 runs (S8 Table). The significance threshold (dashed line) was estimated in 1000 permutations with a 5% cutoff. (E, L) LOD scores of significant QTLs in multiple QTL mapping pipeline (fitqtl). Black lines: additive QTLs, gray lines: QTLs in epistasis. p-value of F statistic is marked: * p-value < 0.05; ** p-value of <0.01. Fitqlt statistics are given in S8 Table. (F, M) Confidence intervals and for the full QTL analysis. QTL intervals: composite interval mapping–orange, scanone–green, fitqtl: additive–black, fitqtl: epistatic–gray, EM-algorithm–blue (exact coordinates of the markers in S8 Table. (G, N): Phenotype panels for the corresponding QTL analysis. The probability of being sensitive (white) or insensitive (black) is shown for each individual. Numbers on the right indicate for each EM panel how many out of 1000 runs that panel was found, and the fraction of individuals in each panel which had an error > 0.10 from the original data (see methods QTL mapping/EM-pipeline, S8 Table). The green marks the panel with the highest convergence and lowest error. (PDF) [file pgen.1010763.s017.pdf]

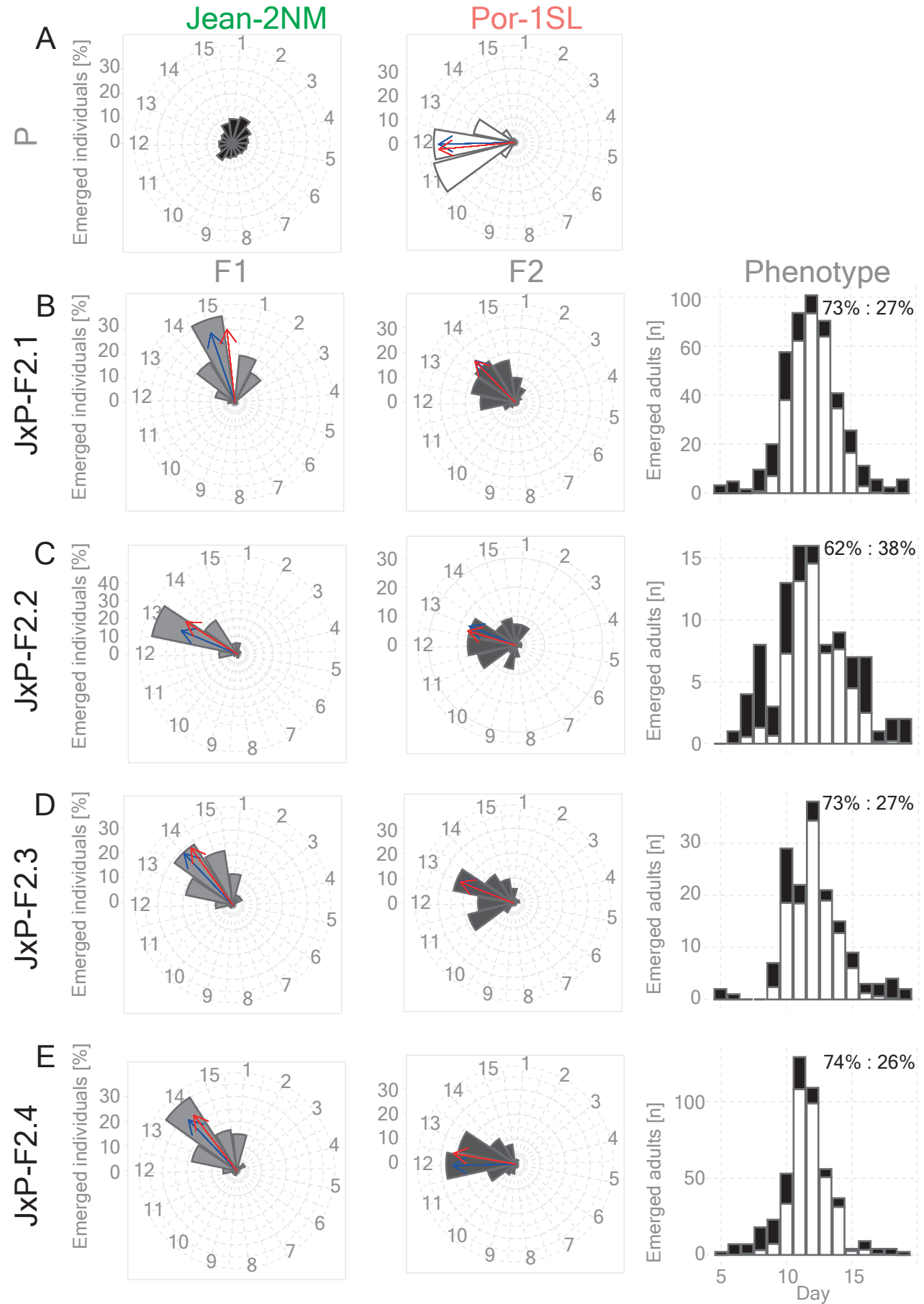

S.Figure 18

Supplement: S18 Fig — (A-E left and middle) The fraction of emerged adults per generation is shown on a circular plot together with the mean and median vector. (A). Jean-2NM parental strain (B) Por-1SL parental strain. (B-E left) Independent F1 progenies of the four intercross families JxP-F2.1–4. (B-E middle) Combined emergence of several F2 families of four intercross families JxP-F2.1–4. Number of individuals is given in S2 Table. (B-E right) Bar graphs show probabilities of finding sensitive Por-1SL-like (white) or insensitive Jean-2NM-like (black) individuals on each day in the four intercross families. (PDF) [file pgen.1010763.s018.pdf]

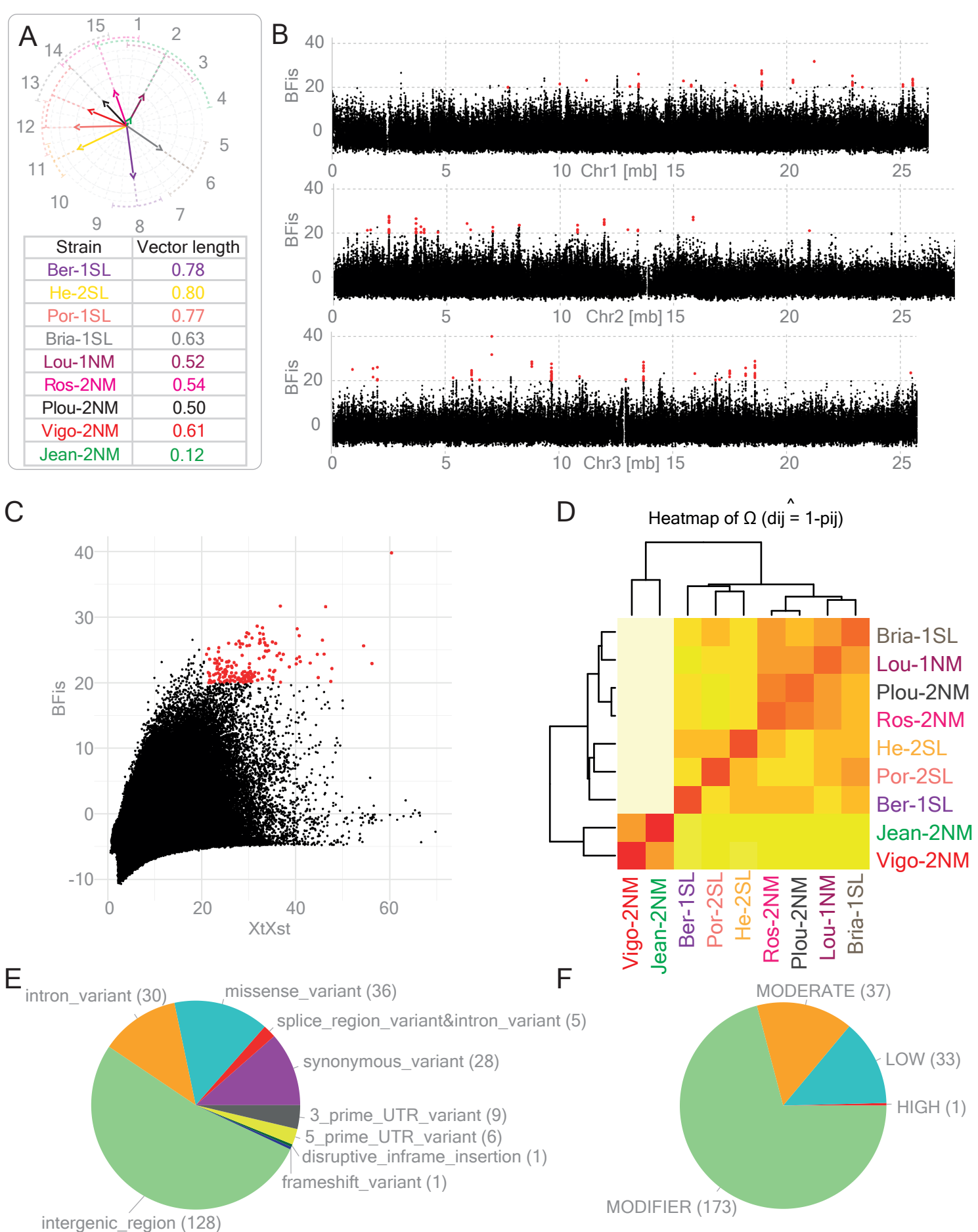

S.Figure 19

Supplement: S19 Fig — Association analysis was performed to find mutations associated with the loss of sensitivity to tidal turbulence in the Jean-2NM population. (A) Median vector length was used as a proxy for sensitivity to this cue (S1 and S5 Tables), (solid lines with arrows; values outside the circle). (B) Association analysis for median vector length with 769.379 SNPs and small indels. Bayesian factor (BFis) is plotted for each variant along the three chromosomes. (C) We found 173 significantly associated SNPs and indels (BFis > 20, eBPis > 2, XtXst > 20.02; see Methods section for details) marked in red. A list of effects and genes affected by mutations is given in S9 Table. (D) Kinship matrix Ω is given as a heatmap showing reconstructed relationships between nine tested populations. (E-F) The effect of 173 associated variants was analyzed by SNPeff. (E) The effects of the variants on the surrounding genes are depicted in a pie chart. (F) The estimated impact of 173 associated variants is represented in a pie chart. (PDF) [file pgen.1010763.s019.pdf]
