# Supplementary material for: Genetic analysis of a phenotypic loss in the mechanosensory entrainment of a circalunar clock
Source: PLoS Genet. 2023 Jun 22;19(6):e1010763. doi: 10.1371/journal.pgen.1010763 (PMC10286985; doi:10.1371/journal.pgen.1010763)
Supplement: S3 Fig — Left: Phenotyping strategy. Light and vibration were logged throughout the experiment and used to calculate the first day of the entrainment as the day when vibration starts in the middle of their subjective night. Emerged adults were collected and the number of emerged individuals per day was recorded. Circular summary statistics were used to test if there was a phase-shift in emergence rhythms between parental, F1, F2, and BC generations. If there was a phase shift, emergence days were corrected so that the only phenotype assessed is rhythmicity. To generalize the emergence distributions, kernel density estimates were calculated (bandwidth = 10) for each generation. The probability of finding sensitive and insensitive individuals on each experimental day in F2 or BC progenies was calculated according to the given equations. The probability of finding an insensitive individual was used as a phenotypic score. In addition, a reduced dataset was generated by removing individuals with uncertain phenotypes between 0.3 and 0.7. Remaining individuals with probability phenotypes > 0.7 or < 0.3 were given binary phenotypes 1 and 0 respectively. Right: QTL mapping strategy. Several mapping pipelines were tested to examine additive and epistatic QTLs. Full and reduced datasets of each crossing family were analyzed using interval mapping (scanone and scantwo), composite interval mapping (WinQTL cartographer), and multiple QTL mapping (fitqtl and qtlbim). See methods section QTL mapping. (PDF) [file pgen.1010763.s003.pdf]

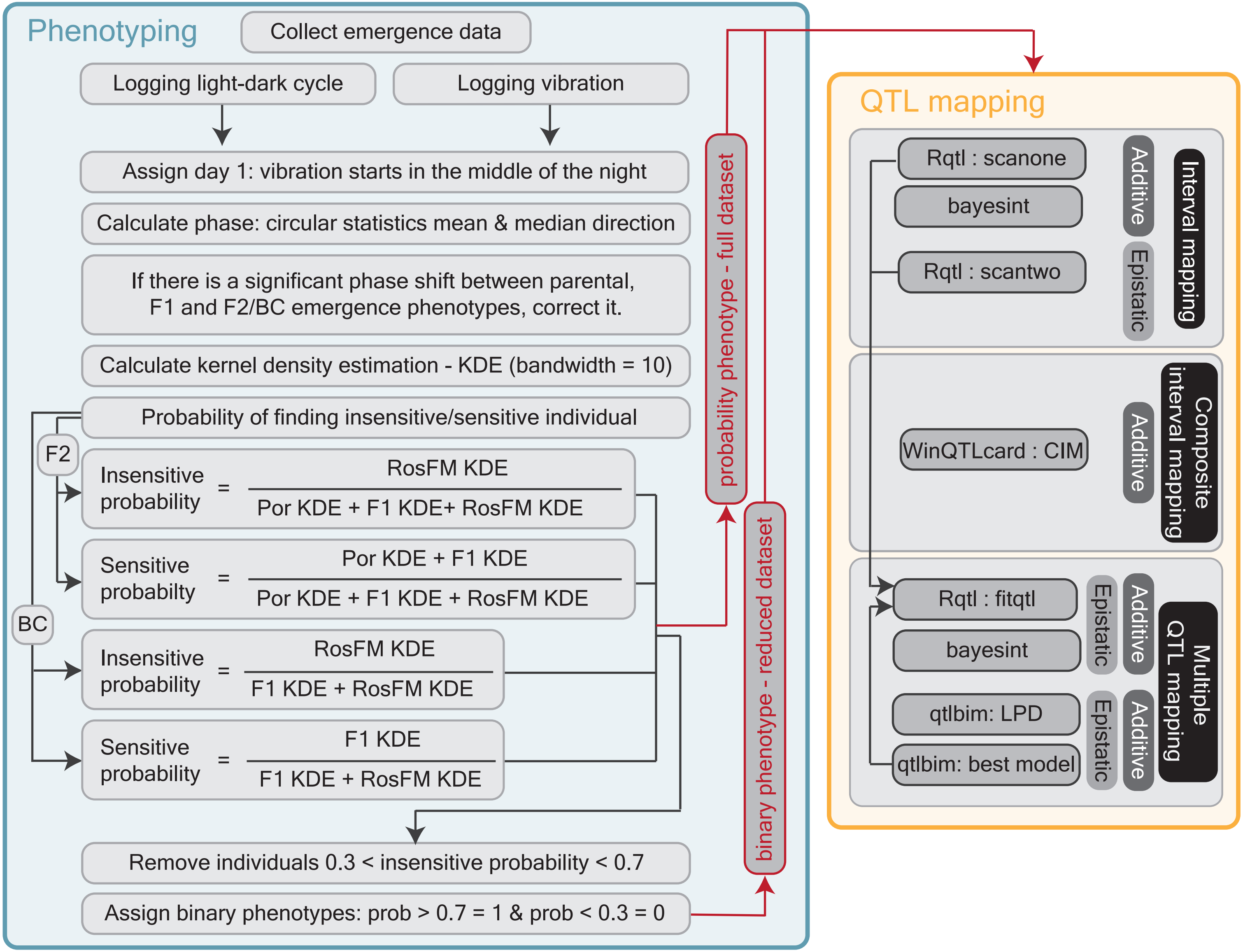

Phenotyping
Collect emergence data
Logging light-dark cycle
Logging vibration
Assign day 1: vibration starts in the middle of the night
Calculate phase: circular statistics mean & median direction
If there is a significant phase shift between parental, F1 and F2/BC emergence phenotypes, correct it.
Calculate kernel density estimation - KDE (bandwidth = 10)
Probability of finding insensitive/sensitive individual
F2
Insensitive probability = RosFM KDE / Por KDE + F1 KDE+ RosFM KDE
Sensitive probabilty = Por KDE + F1 KDE / Por KDE + F1 KDE + RosFM KDE
BC
Insensitive probability = RosFM KDE / F1 KDE + RosFM KDE
Sensitive probability = F1 KDE / F1 KDE + RosFM KDE
Remove individuals 0.3 < insensitive probability < 0.7
Assign binary phenotypes: prob > 0.7 = 1 & prob < 0.3 = 0
probability phenotype - full dataset
binary phenotype - reduced dataset
QTL mapping
Rqtl : scanone
bayesint
Rqtl : scantwo
Additive
Epistatic
Interval mapping
WinQTLcard : CIM
Additive
Composite interval mapping
Rqtl : fitqtl
bayesint
qtlbim: LPD
qtlbim: best model
Epistatic
Additive
Epistatic
Additive
Multiple QTL mapping

S.Figure 3
