# Supplementary material for: Genetic analysis of a phenotypic loss in the mechanosensory entrainment of a circalunar clock
Source: PLoS Genet. 2023 Jun 22;19(6):e1010763. doi: 10.1371/journal.pgen.1010763 (PMC10286985; doi:10.1371/journal.pgen.1010763)
Supplement: S15 Fig — BayPass and SNPeff were used to identify 178 genes associated with the loss of sensitivity to tidal turbulence in Ros-2FM. (A) 51 genes that are driving 78 significant GO terms are depicted. Hierarchical clustering of genes and GO terms reveals major clusters of GO terms (color-coded) and listed in panel (B): yellow = Reproduction, orange = Development & Morphogenesis, brown = Protein & organelle localization, pink = Nervous system, purple = Sensory system, light green = Signaling, dark blue = Circadian, light blue = Metabolic process, dark green = Response, gray = Behavior. (C) Several GO terms related to sensory nervous system and potentially involved in mechanosensory entrainment are given in the table together with the corresponding genes. (D) Venn diagram is showing the number of genes that went into the GO term enrichment analysis. (PDF) [file pgen.1010763.s015.pdf]

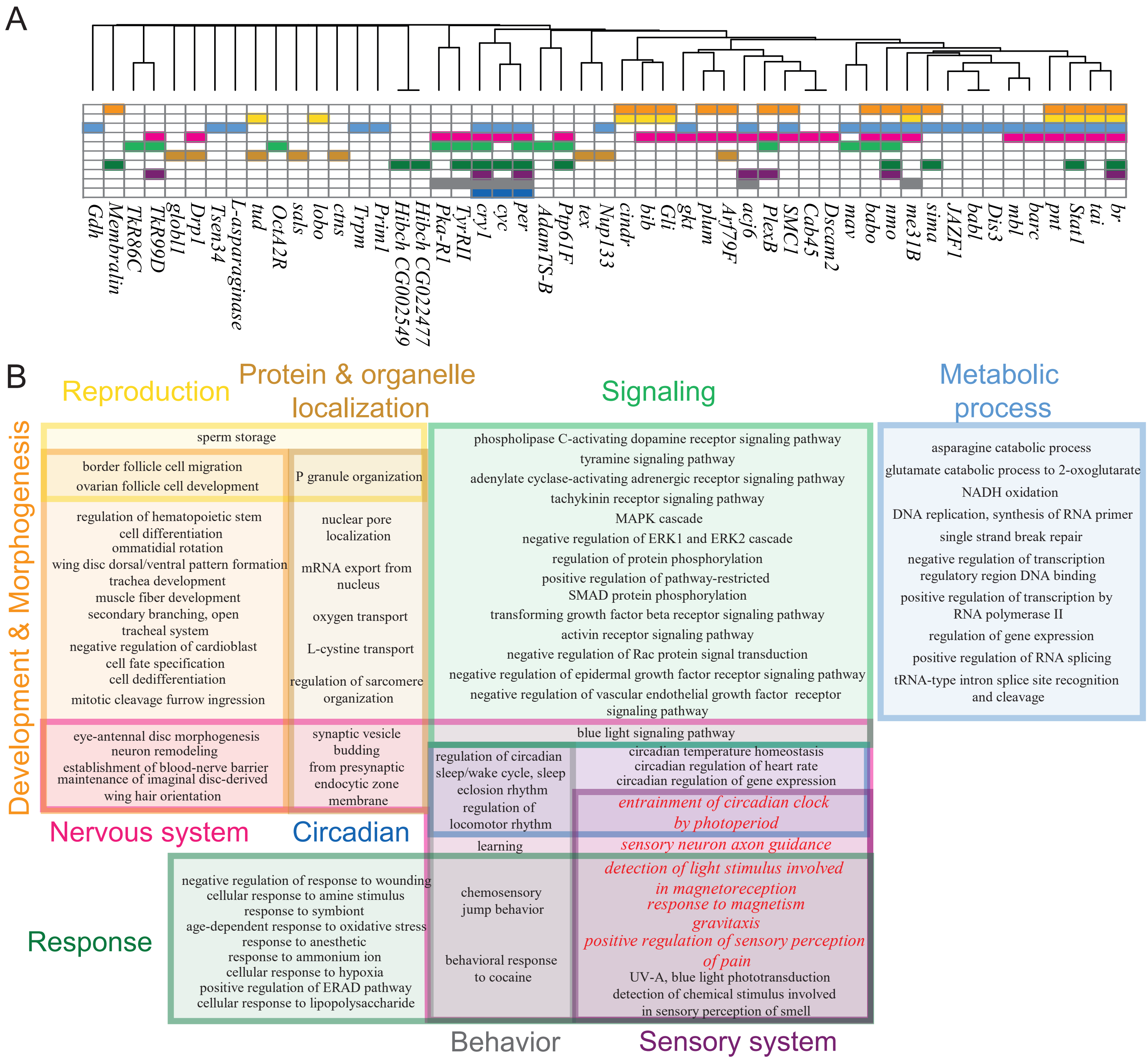

C

GO term	Genes
entrainment of circadian clock by photoperiod	***cry1, per***
sensory neuron axon guidance	***plexin-B***
detection of light stimulus involved in magnetoreception	***cry1***
response to magnetism	
gravitaxis	***cry1, broad, nemo***
positive regulation of sensory perception of pain	***TkR99D***

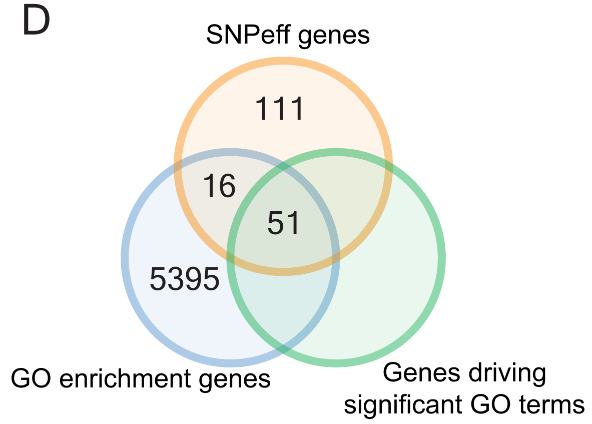

S.Figure 15
