# Supplementary material for: Genetic analysis of a phenotypic loss in the mechanosensory entrainment of a circalunar clock
Source: PLoS Genet. 2023 Jun 22;19(6):e1010763. doi: 10.1371/journal.pgen.1010763 (PMC10286985; doi:10.1371/journal.pgen.1010763)
Supplement: S1 Methods — (DOCX) [file pgen.1010763.s036.docx]

**SUPPLEMENTAL METHODS**

**Genetic analysis of a phenotypic loss in the mechanosensory entrainment of a circalunar clock**

Dušica Briševac^1^, Celine Prakash^1^, Tobias S. Kaiser^1^*

^1^ Max Planck Institute for Evolutionary Biology, Max Planck Research Group Biological Clocks, Plön, Germany

*[kaiser@evolbio.mpg.de](mailto:kaiser@evolbio.mpg.de)

**Contents**

[**Crosses** 1](#_Toc133759690)

[**QTL mapping**: Phenotyping 2](#_Toc133759691)

[**QTL mapping**: Genotyping 5](#_Toc133759692)

[**QTL mapping:** Informative variants and genotype matrix in Jean-2NM x Por-1SL 6](#_Toc133759693)

[**QTL mapping:** Expectation-maximization (EM) algorithm 7](#_Toc133759694)

[**Selective sweep analysis** 8](#_Toc133759695)

[References 10](#_Toc133759696)

# **Crosses**

Due to the genetically encoded differences in the phase of emergence [1], different strains emerge at different times of the day and month when entrained by the same light-dark (LD) regime. Thus, the three strains had to be kept under different LD and artificial moonlight regimes to synchronize the emergence of adults. Ros-2FM and Por-1SL cultures were kept under the same moonlight entrainment and different LD conditions, while Jean-2NM cultures were kept under alternative moonlight and LD regime. Virgin males and females were caught and placed together in a 10x10x5cm box with seawater and kept overnight to allow for mating and oviposition of eggs. On the next day, adults were collected in 100% ethanol and egg clutches in seawater. Egg clutches with over 30 fertilized eggs were raised individually in 10x10x5cm boxes under tidal turbulence entrainment. Emergence patterns in the F1 generation were recorded each day and adults were stored in 100% ethanol. Adults emerging on the same day freely mated and the resulting F2 offspring were raised in individual boxes. Backcrosses were performed by mating F1 adults with Ros-2FM (or Jean-2NM) adults raised under a moonlight regime. To maximize the chance of getting enough synchronized adults for backcrosses, some F1 cultures were also raised under the moonlight regime (S2 Table). Although we tried to generate crosses with both males and females of sensitive and insensitive strains, only crosses between insensitive females and Por-1SL males gave viable offspring (S2 Table). Neumann argued that there may be non-reciprocal cross-sterility in certain Clunio populations [2], but since we did not test this systematically we cannot say if this is indeed the case here or if we got this inequality by chance.

# **QTL mapping**: Phenotyping

Emergence data was collected for parental, F1 and F2, and BC generations, and lunar emergence days under turbulence entrainment were assigned as described above (S2 Table and S1 and S2 Figs). As expected from previous crossing experiments [1,3], a considerable phase-shift of the emergence peaks was sometimes found between F1, F2/BC families, and the rhythmic Por-1SL parental strain (S2 Table and S2, S4, S15, and S16 Figs). Since the phase is genetically determined [1,3], we corrected for the phase shift to not mistakenly identify QTLs encoding phase. We first calculated the phase of the peaks in the parental populations as well as F1, F2, and BC families, and if we found a phase difference, we then reassigned the “days under tidal turbulence entrainment” of F2/BC so that the peak days match the Por-1SL peak days (S2 Table and S4 and S16 Figs). More specifically, we used the R package “circular” [4] and calculated the vector mean direction *mean.circular,* vector median direction *median.circular*, direction length *rho.circular,* sample circular standard deviation *sd.circular,* and angular variance *angular.variance* (S1 Script and S2 Table). Due to the big differences in the total numbers of individuals collected in parental strains (S1 Table) and F1/F2/BC families (S2 Table) we did not perform statistical tests designed to assess if the distributions were significant. According to the mean and median directions, peak days in the RPxR-BC.1 family remained unchanged while RxP-F2.1, JxP-F2.1.6, and JxP-F2.2.3 families were shifted by 2 days (Table 1 and S4 and S16 Figs).

In order to phenotype sensitivity to tidal turbulence, we must distinguish between “sensitive” individuals that emerged within the Por-1SL-like peak and “insensitive” that can emerge on any lunar day. A phenotyping problem arises because the emergence peak does not only contain sensitive (rhythmic) individuals, but also some of the insensitive (arrhythmic) individuals. Hence, for individuals found in the emergence peak, we do not know a priori which phenotype they were, while individuals outside the emergence peak are certainly insensitive. To resolve this problem of overlapping phenotypes, we designed a pipeline to calculate the probability of finding “sensitive” and “insensitive” individuals on each day. To get an emergence estimate from raw emergence distributions we calculated kernels density estimates using a *density.circular(bw = 10)* function from R package “circular”. We then calculated the average kernel’s density estimate for each day for parental, F1, F2, and BC distributions. The probabilities of finding sensitive and insensitive individuals on a given day were calculated the following way.

Intercross:

Probability of being insensitive = insensitive parent density / (sensitive parent density + F1 density + insensitive parent density)

Probability of being sensitive = (sensitive parent density + F1 density) / (sensitive parent density + F1 density + insensitive parent density)

Backcross:

Probability of being insensitive = insensitive parent density / (F1 density + insensitive parent density)

Probability of being sensitive = F1 density / (F1 density + insensitive parent density)

We found that sensitivity to turbulence is inherited dominantly. As a result, in the second generation of an intercross family, sensitive individuals have either Por-1SL or F1 genotypes, while insensitive individuals have Ros-2FM or Jean-2NM genotypes. Furthermore, in a back-cross family, the sensitive individuals have F1 genotypes and insensitive individuals have Ros-2FM or Jean-2NM genotypes. The probability of being insensitive was used as a phenotypic score for QTL mapping. In addition, we noticed that on certain days the probability of finding sensitive and insensitive individuals is almost equal, andthe individuals that emerged on the day when the probability is 0.5 introduce the most error in the analysis To test how much those individuals influence mapping results, we generated reduced datasets by excluding individuals with probability phenotypes of 0.5+- 0.2, and assigning binary phenotypes to the remaining ones: probability > 0.7 gained phenotype 1, probability < 0.3 gained phenotype 0 (S1 Script and S3 Fig). We tried different cut-offs as well, but with 0.5 +- 0.3 or more, the borders became too stringent and we were left with too few individuals for the QTL mapping to be reliable. In other words, the cutoffs of 0.7 and 0.3 were the values with which we could remove the most “uncertain” individuals and still have enough individuals remaining for the QTL analysis to be reliable.

# **QTL mapping**: Genotyping

DNA was extracted from adults collected in crossing experiments with the salting-out method [5]. Genomic DNA was amplified with standard RepliG protocol (REPLI-g Mini Kit QIAgen 150025). Single-digest RAD sequencing was used to sequence the RPxR-BC.1 family [6]. In brief, 100ng of DNA per sample was digested with BamHI-HF (NEB) at 37°C for 2 h, P1 adapters with BamHI sticky ends (S10 Table) were ligated with T4 Ligase (NEB) at room temperature for 1h, and heat-inactivated at 65°C for 20 minutes. Samples labelled with unique P1 adapters were pooled and sheared by sonication (Covaris S220 focused-ultrasonicator: Duty cycle = 10; Intensity = 7; Cycles/burst = 300). Following sonication, DNA was precipitated, loaded on the 1% TBE gel, and fragments of the size between 300 and 700bp were extracted from the gel (Zymoclean™ Gel DNA Recovery Kit). Quick Blunting Kit (NEB) was then applied to polish DNA ends and Klenow exo (NEB) to add A-overhangs. Finally, P2 adapters (S10 Table) were ligated with T4 ligase (NEB), and DNA was amplified with Phusion Master Mix (HF) using P1/P2 amplification primers (S10 Table) in 12 PCR cycles. Sequencing was performed on Illumina HiSeq3000 with single-end 150 bp reads. To genotype RxP-F2.1, JxP-F2.1.6, and JxP-F2.2.3 families, the double-digest RAD sequencing protocol was optimized [7,8]. Briefly, 100 ng of DNA per individual was digested with BglII (#FD0084 Thermo Fisher) and MspI (#FD0544 Thermo Fisher) for 2h 37°C. P1 and P2 adapters (S10 Table) were ligated simultaneously with T4 ligase (#EK0032 Thermo Fisher) for 2 hours at 22°C and heat-inactivated at 70°C for 10min. Samples were pooled, precipitated, and loaded on the 1% TEB gel, and DNA fragments sizes between 200 and 1000bp were extracted (Zymoclean™ Gel DNA Recovery Kit) and amplified with Phusion Master Mix (HF) using P1/P2 amplification primers (S10 Table) in 12 PCR cycles. Sequencing was performed on Illumina HiSeq3000 with single-end reads for RPxR-BC.1 family and paired-end for RxP-F2.1, JxP-F2.1.6, and JxP-F2.2.3 families.

# **QTL mapping:** Informative variants and genotype matrix in Jean-2NM x Por-1SL

Samples from parents’ and F1s of the two Jean-2NMxPor-1SL families, unfortunately, had very few good genotypes. Thus, we designed an alternative approach for reconstructing the recombination matrix. RAD sequencing VCF files were filtered for minGQ 20, max-alleles 2, and max-missing 0.60. The few genotypes for which we had parents’ and F1’s genotypes were used as fixed guides (the same coding strategy as in S11 Table). The remaining markers were kept if they had genotypes in the Jean-2NM and Por-1SL pool-sequencing data generated by sequencing 300 individuals of each strain [3]. We kept markers that in F2 offspring had all 3 genotypes (0/0, 0/1, 1/1) and follow the Hardy–Weinberg principle: 0/0 > 10% or 1/1 > 10% or 0/1 > 40% (Hardy 1908). We then identified recombination events based on genotype-switching along the chromosomes from heterozygous to either of the two homozygous genotypes because we knew that 0/1 is certainly AB but 0/0 and 1/1 could have been either AA or BB depending on the genotypes of the parents (see S11 Table). We resolved the homozygous genotypes by using parents’ genotypes we had and the consistency genotype assignment in S2 Script. Individuals with no genotypes were excluded. Since the two parent strains are fairly divergent, we used the markers’ allele frequency in the natural populations to compare the resolved parent genotypes. As a final quality control, we ran a QTL pipeline using sex as a phenotype since its confidence interval is known [3]. The results were as expected (S10 Fig and S12 Table), so we are confident that the recombination matrix was correctly reconstructed. The final number of markers was 560 in JxP-F2.1.6 and 178 in the JxP-F2.2.3 family. The final genotype matrix is given in S8 Table.

# **QTL mapping:** Expectation-maximization (EM) algorithm

To explore the effect of uncertainty in phenotyping on the QTL mapping results, we devised an EM algorithm to assign binary phenotypes to the entire dataset (S5 Fig). We started from calculated insensitivity probabilities, ran the *scanone* function of the R/qtl package [9], and recorded the maximal log odds ratio (LOD) score. We then assigned binary phenotypes to all individuals considering their given insensitivity probabilities. This implies that individuals with probabilities of 0 or 1 could not change. Individuals with an insensitivity probability of say 0.3 would have a 30% chance of being recorded as insensitive (0) and a 70% chance of being recorded as sensitive (1). On the resulting binary phenotype set, we ran *scanone* again and recorded the maximal LOD score. This was repeated 10.000 times and the iteration that gave the highest LOD score overall was kept. In a second step, starting from the best binary phenotype panel we systematically flipped phenotypes of one individual at the time and kept only the single flipped score which increased the maximal LOD score the most. This was iterated until there was no further improvement (maximally 200 times), driving the binary phenotype panel into a local optimum for the LOD score. We ran the entire algorithm with both steps 1000 times from the start and each final optimized phenotype panel was recorded. We then calculated in how many of the 1000 iterations the same optimal panel was found, and how much the optimized binary phenotypes differed from the starting probabilities:

Error per individual in EM panel = ABS (probability of being insensitive – binary phenotype) x ABS (0.5 - the probability of being insensitive)

To assess how much QTL results based on starting probabilities match the results from EM binary phenotypes we looked at 1) the percentage of convergence in 1000 EM loops and 2) the percentage of individuals in a binary panel with error > 0.10. QTL intervals were determined for the final optimized panels that appeared in more than 5% of the 1000 runs.

# **Selective sweep analysis**

We used selscan 2.0 to calculate XP-nSL [10,11]. Selscan 2.0 assumes that the data is polarized (ancestral and derived state of the alleles is known) and we do not have an outgroup or an ancestral DNA. Thus, we used the major allele in the entire dataset that consists of 15 populations and 349 individuals (20-24 per population) as the ancestral allele. The vcf file containing GATK-called SNPs and indels from 349 individuals was filtered for biallelic sites (leaving 7.134.648 variants) with vcftools version 0.1.14 [12] and then polarized the dataset by setting 0 = major (ancestral) allele and 1 = minor (derived) allele the following way. Vcf files were converted to plink input files with vcftools, allele frequency was calculated for A1 (minor) and A2 (major) alleles in plink, with *--freq* parameter, and a text file was outputted that contains 2 columns: the identity of the major allele and the variant position, which is then used in plink to set the major allele as 0 with *--a1-allele*.

Finally, to search for loci that sweep through the turbulence-insensitive populations due to local adaptation, we contrasted Ros-2FM and Jean-2NM with the closest turbulence-sensitive populations: Ros-2NM and Vigo-2NM respectively. A vcf file consisting of 48 Ros-2FM and Ros-2NM individuals were filtered for minor allele frequency = 0.05, no missing data, and minimal quality (minQ) of 20 leaving 574.132 variants. Similarly, the vcf file containing 47 Jean-2NM and Vigo-2NM males was filtered the same way leaving 697.594 variants.

We calculated XP-nSL per chromosome with *selscan2.0* *--unphased* keeping the default settings: *--cutoff* (the EHH decay cut off) of 0.05; *--gap-scale* (if a gap is encountered between two SNPs the genetic distance is scalled by GAP_SCALE/GAP) was 20.000; *--max-extend* (the maximum distance the EHH decay curve is allowed to extend from the core) was 1.000.000; *--max-gap* (maximum allowed gap in bp between two SNPs) was 200.000. We then ran the *norm* to calculate normalized XP-nSL for all chromosomes together and to identify top candidate regions that contain a significantly high number of core alleles in a given window. We kept default parameters --*crit-val* (iHS based on [13]) of 2, *--min-SNPs* (only consider windows with at least this many SNPs) of 10, *--qbins* (the number of quantile bins to use when identifying significant windows binned by a number of sites within each) of 10. We changed the window size *--winsize* from the default value of 100.000 to 10.000 bp because the *Clunio* genome is much smaller than the *Human* genome on which the tool was optimized. In addition, the clusters of SNPs with high association values we identified thus far do not exceed 10kb (See S6 and S9 Tables).

# References

1. Kaiser TS, Heckel DG. Genetic architecture of local adaptation in lunar and diurnal emergence times of the marine midge clunio marinus (chironomidae, diptera). PLoS One. 2012;7. doi:10.1371/journal.pone.0032092

2. Neumann D. Eine nicht-reziproke Kreuzungssterilität zwischen ökologischen Rassen der Mücke Clunio marinus. Oecologia (Berl). 1971;8: 1–20.

3. Kaiser TS, Poehn B, Szkiba D, Preussner M, Sedlazeck FJ, Zrim A, et al. The genomic basis of circadian and circalunar timing adaptations in a midge. Nature. 2016;540: 69–73. doi:10.1038/NATURE20151

4. Pewsey Arthur, Neuhaeuser Markus, Ruxton D. Graeme. Circular Statistics in R. Oxford University Press; 2013.

5. Reineke A, Karlovsky P, Zebitz CPW. Preparation and purification of DNA from insects for AFLP analysis. Insect Mol Biol. 1998;7: 95–99.

6. Baird NA, Etter PD, Atwood TS, Currey MC, Shiver AL, Lewis ZA, et al. Rapid SNP discovery and genetic mapping using sequenced RAD markers. PLoS One. 2008;3. doi:10.1371/journal.pone.0003376

7. Etter PD, Bassham S, Hohenlohe PA, Johnson EA, Cresko WA. SNP discovery and genotyping for evolutionary genetics using RAD sequencing. Methods Mol Biol. 2011;772: 157–178. doi:10.1007/978-1-61779-228-1_9

8. Etter PD, Johnson E. RAD paired-end sequencing for local de novo assembly and SNP discovery in non-model organisms. Methods in Molecular Biology. 2012;888: 135–151. doi:10.1007/978-1-61779-870-2_9

9. Karl W. Broman, Saunak Sen. A Guide to QTL Mapping with R/qtl. Springer; 2009. doi:10.1007/978-0-387-92125-9

10. DeGiorgio M, Szpiech ZA. A spatially aware likelihood test to detect sweeps from haplotype distributions. bioRxiv. 2021; 2021.05.12.443825. doi:10.1101/2021.05.12.443825

11. Szpiech ZA, Novak TE, Bailey NP, Stevison LS. Application of a novel haplotype-based scan for local adaptation to study high-altitude adaptation in rhesus macaques. Evol Lett. 2021;5: 408–421. doi:10.1002/EVL3.232

12. Danecek P, Auton A, Abecasis G, Albers CA, Banks E, DePristo MA, et al. The variant call format and VCFtools. Bioinformatics. 2011;27: 2156–2158. doi:10.1093/bioinformatics/btr330

13. Voight BF, Kudaravalli S, Wen X, Pritchard JK. A Map of Recent Positive Selection in the Human Genome. PLoS Biol. 2006;4: e72. doi:10.1371/JOURNAL.PBIO.0040072
