# Supplementary material for: Genetic analysis of a phenotypic loss in the mechanosensory entrainment of a circalunar clock
Source: PLoS Genet. 2023 Jun 22;19(6):e1010763. doi: 10.1371/journal.pgen.1010763 (PMC10286985; doi:10.1371/journal.pgen.1010763)
Supplement: S10 Fig — To complement the S9 Fig. and validate the resolved Jean-2NMxPor-1SL genotype matrix (see S1 Methods: QTL mapping: Informative variants and genotype matrix in Jean-2NM x Por-1SL), we identified the sex locus in the four mapping families: (A)RPxR-BC.1, (B) (RxP-F2.1, (C) JxP JxP-F2.1.6, (D) JxP-F2.2 .3. The QTL intervals are listed in S10 Table. (PDF) [file pgen.1010763.s010.pdf]

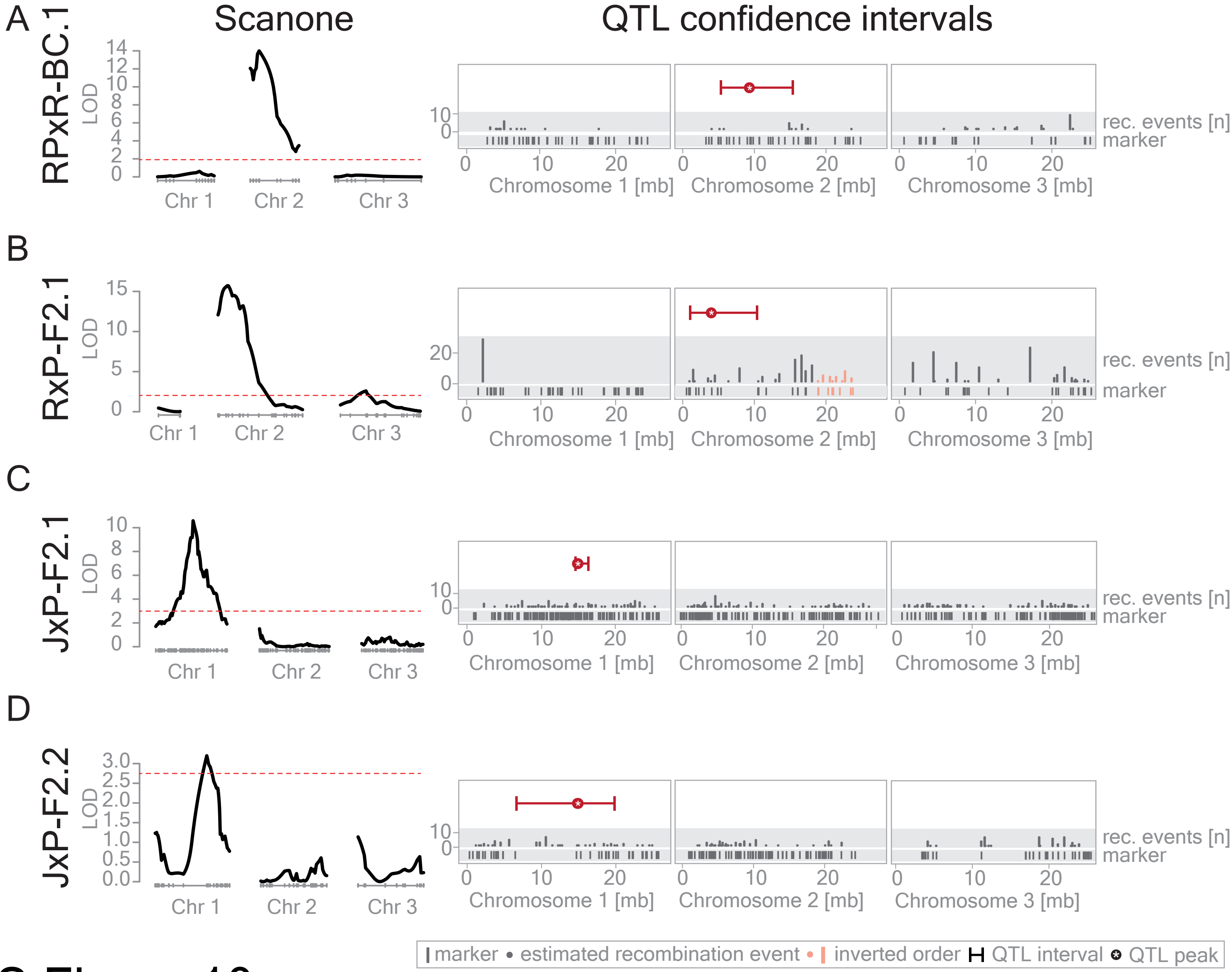
A
B
C
D
Scanone
QTL confidence intervals
RPxR-BC.1
RxP-F2.1
JxP-F2.1
JxP-F2.2
LOD
Chr 1
Chr 2
Chr 3
Chromosome 1 [mb]
Chromosome 2 [mb]
Chromosome 3 [mb]
rec. events [n]
marker
marker • estimated recombination event • inverted order H QTL interval QTL peak

S.Figure 10
